# The therapeutic mechanism of Curcumae Radix against primary dysmenorrea based on 5-HTR/Ca^2+^/MAPK and fatty acids metabolomics

**DOI:** 10.3389/fphar.2023.1087654

**Published:** 2023-03-09

**Authors:** Yuwen Qin, Wei Zhang, Zhenhua Bian, Chenghao Fei, Lianlin Su, Rong Xue, Qian Zhang, Yu Li, Peng Chen, Yabo Shi, Mingxuan Li, Chunqin Mao, Xiaoli Zhao, De Ji, Tulin Lu

**Affiliations:** ^1^ College of Pharmacy, Nanjing University of Chinese Medicine, Nanjing, China; ^2^ College of Pharmacy, Anhui University of Chinese Medicine, Hefei, China; ^3^ Anhui Province Key Laboratory of Traditional Chinese Medicine Decoction Pieces of New Manufacturing Technology, Hefei, China; ^4^ Department of Pharmacy, Wuxi Traditional Chinese Medicine Hospital Affiliated to Nanjing University of Chinese Medicine, Wuxi, China; ^5^ Jiangsu Provincial Engineering Research Center of TCM External Medication Development and Application, Nanjing University of Chinese Medicine, Nanjing, China; ^6^ State Administration of Traditional Chinese Medicine: Traditional Chinese Medicine Concoction Technology Inheritance Base, China

**Keywords:** curcuma radix, serum pharmacochemistry, isolated uterine spasm model, FA metabolism, 5-HTR/Ca^2+^ /MAPK, primary dysmenorrea

## Abstract

**Background:** *Curcumae* Radix (CW) is traditionally used to treat primary dysmenorrea (PD). However, the mechanisms of action of CW in the treatment of PD have not yet been comprehensively resolved.

**Objective:** To investigate the therapeutic effects of CW on PD and its possible mechanisms of action.

**Methods:** An isolated uterine spastic contraction model induced by oxytocin was constructed in an *in vitro* pharmacodynamic assay. An animal model of PD induced by combined estradiol benzoate and adrenaline hydrochloride-assisted stimulation was established. After oral administration of CW, a histopathological examination was performed and biochemical factor levels were measured to evaluate the therapeutic effect of CW on PD. The chemical compositions of the drug-containing serum and its metabolites were analyzed by ultra-high-performance liquid chromatography coupled with quadrupole time-of-flight tandem mass spectrometry. Network pharmacology and serum untargeted metabolomics were used to predict the mechanism of CW treatment for PD, and the predicted results were validated by RT-qPCR, WB, and targeted fatty acid (FA) metabolism.

**Results:** *In vitro,* CW can relax an isolated uterus by reducing uterine motility. *In vivo*, the results showed that CW attenuated histopathological damage in the uterus and regulated PGF_2α_, PGE_2_, β-EP, 5-HT, and Ca^2+^ levels in PD rats. A total of 66 compounds and their metabolites were identified in the drug-containing serum, and the metabolic pathways of these components mainly included hydrogenation and oxidation. Mechanistic studies showed that CW downregulated the expression of key genes in the 5-HTR/Ca^2+^/MAPK pathway, such as 5-HTR2A, IP3R, PKC, cALM, and ERK. Similarly, CW downregulated the expression of key proteins in the 5-HTR/Ca^2+^/MAPK pathway, such as p-ERK/ERK. Indirectly, it ameliorates the abnormal FA metabolism downstream of this signaling pathway in PD rats, especially the metabolism of arachidonic acid (AA).

**Conclusion:** The development of PD may be associated with the inhibition of the 5-HTR/Ca^2+^/MAPK signaling pathway and FA metabolic pathways, providing a basis for the subsequent exploitation of CW.

## 1 Introduction

Primary dysmenorrea (PD) is common, occurring in 50%–90% of adolescent girls and women of childbearing age, seriously affecting women’s work and studies, which could reduce their quality of life (Mckenna and Fogleman, 2021). According to reports, primary dysmenorrea is related to endocrine disorders and pressining or mental factors, such as tension, anxiety, depression, or poor lifestyle habits, which lead to excessive contraction of the uterine muscles and arterial vasoconstriction, resulting in uterine ischemia and pain (Sultan et al., 2012; Szmidt et al., 2020). Currently, most pain-relieving and sedative drugs, such as non-steroidal anti-inflammatory drugs (NSAIDs), are used to treat PD, such as ibuprofen (IBP), which can relieve the patient’s pain but cannot cure it and has strong drug dependence and many side effects. In contrast, the use of traditional Chinese medicine (TCM) to treat PD has several clinical advantages (Su et al., 2021; Tong et al., 2021).

*Curcumae* Radix (CW) is the dried root tuber of *Curcuma wenyujin* Y.H.Chen et C. Ling, *Curcuma longa* L, *Curcuma kwangsiensis* S.G.Lee & C.F.Liang and *Curcuma phaeocaulis* Val. (The plant name has been checked with http://www.theplantlist on 7 October 2022) (CHINESE PHARMACOPOEIA COMMISSION, 2020). CW is traditionally used to treat painful inflammatory conditions, such as amenorrhea, irregular menstruation, and dysmenorrea (Chen X. J. et al., 2018; Sahin et al., 2018; CHINESE PHARMACOPOEIA COMMISSION, 2020). Curcumin and germacrone, which are found in CW, have been shown to exert blood-activating effects by reducing blood viscosity, decreasing platelet aggregation, and improving blood rheology (Fang et al., 2019; Tong et al., 2021). This finding also provides a clinical rationale for CW in the treatment of PD. However, the material basis and mechanism of CW in the treatment of PD have not yet been systematically studied. Therefore, it is important to systematically study the potential mechanisms of CW in the treatment of PD.

Given the above problems, this study investigated the potential material basis and mechanism of action of CW in treating PD using classical spectroscopy, network pharmacology, metabolomics, and transcriptomics. First, *in vitro* and *in vivo* drug efficacy experiments were conducted to evaluate. Ultra-high-performance liquid chromatography coupled with quadrupole time-of-flight tandem mass spectrometry (UPLC-Q-TOF-MS/MS) was used to investigate the CW, and the drug-containing serum obtained in animal experiments was rapidly analyzed for its material basis. Based on the detected chemical components, network pharmacology was used to construct PD-related *active ingredients–core targets–critical pathway* regulatory network to predict the potential targets and signaling pathways of CW for the treatment of PD. Serum untargeted metabolomics was used to predict the potential metabolic pathways of CW for PD treatment. Finally, the predicted signaling and metabolic pathways were validated using quantitative real-time fluorescence polymerase chain reaction (RT-qPCR), Western blotting (WB), and targeted FA metabolism to preliminarily elucidate the mechanism of action of CW in the treatment of PD.

## 2 Materials and methods

### 2.1 Medicinal plants, chemicals, and reagents

CW was purchased from Ruian Tongming *C. wenyujin* professional cooperatives (Zhejiang, China). The samples were identified as root tubers of *C. wenyujin* Y.H.Chen et C. Ling by Professor Jianwei Chen at the Nanjing University of Chinese Medicine (NJUCM). Voucher specimens (202106291) were stored at NJUCM. Additional detailed information was provided in Supplementary materials S1.

### 2.2 Study on the efficacy of CW in treating PD

#### 2.2.1 Animals and grouping

Females SPF Sprague-Dawley (SD) rats (200 ± 20 g, certificate No. 20210910Aazz0619000549), which were not pregnant and did not mate, were purchased from Zhejiang Weitonglihua Experimental Animal Technology Co., Ltd. [License No. SCXK (Zhejiang) 2019-0001]. The rats were kept in a constant temperature and humidity environment (25°C ± 2 C, 60% ± 5%), and a 12 h dark/light cycle. All rats were provided with a standard diet and water *ad libitum*.

After 1 week of acclimatization, the rats were divided randomly into ibuprofen positive control group (IBP) (*n* = 6), and CW group (*n* = 6) *in vitro* pharmacodynamics. The rats were divided randomly into a normal control group (NC, *n* = 8), Model group (M, *n* = 8), ibuprofen positive control group (IBP, *n* = 8), CW low-dose group (CW-L, *n* = 8); CW high-dose group (CW-H, *n* = 8) *in vivo* pharmacodynamics. The CW slices and extracts were prepared as described in a study (Tong et al., 2021). Finally, CW extracts containing 0.2 g·mL^−1^ raw medicine were obtained.

#### 2.2.2 *In vitro* pharmacodynamics: An *ex vivo* uterine contraction model

The experimental protocol and the method of administration were as described in this study (Qin et al., 2022), but the extraction method of CW was the difference.

#### 2.2.3 *In vivo* pharmacodynamics: PD model

##### 2.2.3.1 Experimental protocol and drug administration

This method was modified based on our previous research (Chen Z. M. et al., 2018; Tong et al., 2021). Estradiol benzoate was injected subcutaneously into the back of each rat on days 1 and 10 at 5 mg·kg^−1^·d^−1^, and on days 2–9 at 2.5 mg·kg^−1^·d^−1^. Epinephrine hydrochloride was injected at 0.6 mg·kg^−1^·d^−1^ twice daily, with an interval of approximately 4 h, supplemented by combined stimulation (a: acoustic stimulation (60 dB, (10 ± 5) kHz, intensity level 3), 60 min/time, 2 times/day. b: Light stimulation (flickering light, frequency (2 ± 1) Hz), 60 min/time, 2 times/day. c: restraint tube, 60 min/session, 2 times/day. d: clip tails, 30 min/time, 3 times/day). Gastric gavage was started on day 4 and administered once daily for 7 days (Supplementary Figure S1).

Converted to clinically equivalent doses, the dose was 0.06 g·kg^−1^ for the IBP group, 0.3 g·kg^−1^ for the CW-L group, and 0.9 g·kg^−1^ for the CW-H group, and other groups were administered orally with equal amounts of saline daily for 7 days. The CW dosage was defined as the upper and lower limits stipulated in the Chinese Pharmacopoeia (CHINESE PHARMACOPOEIA COMMISSION, 2020).

##### 2.2.3.2 Samples collection and detection

On day 10, 3% sodium pentobarbital (1 mL·kg^−1^) was injected intraperitoneally for anesthesia. The blood was taken, left to stand for 1 h, then centrifuged at 3 000 r·min^−1^ for 20 min, and the serum was collected. The fresh rat uterus and liver tissues were quickly collected, rinsed in saline, and blotted on filter paper. The uterus tissues were fixed with 4% paraformaldehyde; sequentially sectioned by paraffin embedding, dewaxing, and washing; hematoxylin stained, eosin stained, and sealed by dehydration. A Vectra 3.0 fully automated quantitative pathology workstation (PerkinElmer, United States) was used for image acquisition of the sections, and each section was viewed at 20 × 20 to observe all tissues and observe gross lesions. ELISA was used to determine the levels of PGF_2α_, PGE_2_, β-EP, and Ca^2+^ levels in the uterine tissues and the level of 5-HT in the serum. The levels of PGF_2α_, PGE_2_, β-EP, and 5-HT were determined by the competitive method, and the Ca^2+^ level was determined by the biochemical microplate method, which was carried out strictly according to the manufacturer’s instructions.

### 2.3 Study on the effective components of CW in treating PD based on UPLC-Q-TOF-MS/MS

The sample solutions of serum were prepared according to a previously established method (Fei et al., 2022; Qin et al., 2022). In addition, quality control samples were prepared simultaneously to ensure the suitability of the method and system. The mixed stock solutions of 11 standards were prepared (approximately 5 μg/mL). The Liquid chromatographic and mass spectrometric conditions were similar to those used in a previous article (Qin et al., 2022). More details can be found in Supplementary materials S2.

### 2.4 Prediction of the mechanism of CW in the treatment of PD

#### 2.4.1 Network pharmacology analysis

The key targets and signaling pathways of CW for the treatment of PD were predicted by network pharmacology, the active ingredients and core targets were screened by relevant websites and databases, and GO and KEGG enrichment analyses were performed. Using Cytoscape 3.9.1 (http://www.cytoscape.org) software, a network diagram of active components-potential targets-signal pathways was integrated and constructed.

#### 2.4.2 Serum non-targeted metabolomics analysis

The experimental method for serum non-targeted metabolomics was the same as the above-mentioned serum composition method (Fei et al., 2022). The One-MAP cloud platform was used to analyze the of each group (NC groups, M groups, and CW-H groups) serum mass spectrometry data and to identify the differential metabolites among the serum samples, which were compared with the standard secondary mass spectrometry data from the KEGG, HMDB, METLIN, and other compound databases as well as relevant literature. MetaboAnalyst 4.0 was used to perform metabolic pathway enrichment analysis on the differential metabolites (Hao et al., 2018).

### 2.5 Verification of the mechanism of CW in the treatment of PD

#### 2.5.1 Regulation of CW on the 5-HTR/Ca^2+^/MAPK signal pathway of PD

RT-qPCR: Total uterine tissue cellular RNA was extracted and reverse transcribed according to the RNA extraction and reverse transcription kits, respectively. Specific primers were designed based on previous studies (Supplementary Table S1) (Wang et al., 2017; Wu et al., 2019).

WB: Total proteins from the uterine tissues were first extracted and denatured by KZ-III-F tissue homogeniser (Servicebio, China). This was followed by sequential SDS-PAGE electrophoresis, membrane transfer, immunoassay, and chemiluminescence experiments.

More detailed steps can be found in Supplementary materials S3.

#### 2.5.2 Regulation of CW on targeted FA metabolism of PD

Sample pretreatment and the preparation of medium-chain and long-chain FA methyl ester standard solutions were carried out according to the methods of previous studies, and the scanning was carried out under the conditions of gas chromatography and mass spectrometry (Liu et al., 2020)**.** Automatic identification and integration of each ion fragment were performed using v10.0.707.0 Masshunter quantitative software (Agilent, United States) using default parameters and aided by manual inspection. A linear regression standard curve was plotted using the mass spectral peak area of the analyte as the vertical coordinate and the concentration of the analyte as the horizontal coordinate.

Sample concentration calculation: The mass spectral peak areas of the sample analytes were substituted into the linear equation to calculate the concentration results. Sample FA content calculation formula: Sample FA content (μg/mg) = (C × V)/M. C (μg/mL) was the concentration determined by 8890-7000 D GC-MS (Agilent Technologies Inc. UAS); V (mL) is the fixed volume and M (mg) is the sample weight at the time of extraction. More details can be found in Supplementary materials S3.

### 2.6 Statistical analysis

One-way analysis of variance and *t-test* were performed using the GraphPad 8 software (GraphPad Software Inc, United States) (‾x ± s; *p* < 0.05).

## 3 Results

### 3.1 Efficacy of CW in treating PD

#### 3.1.1 *In vitro* pharmacodynamics: An *ex vivo* uterine contraction model

**FIGURE 1:**
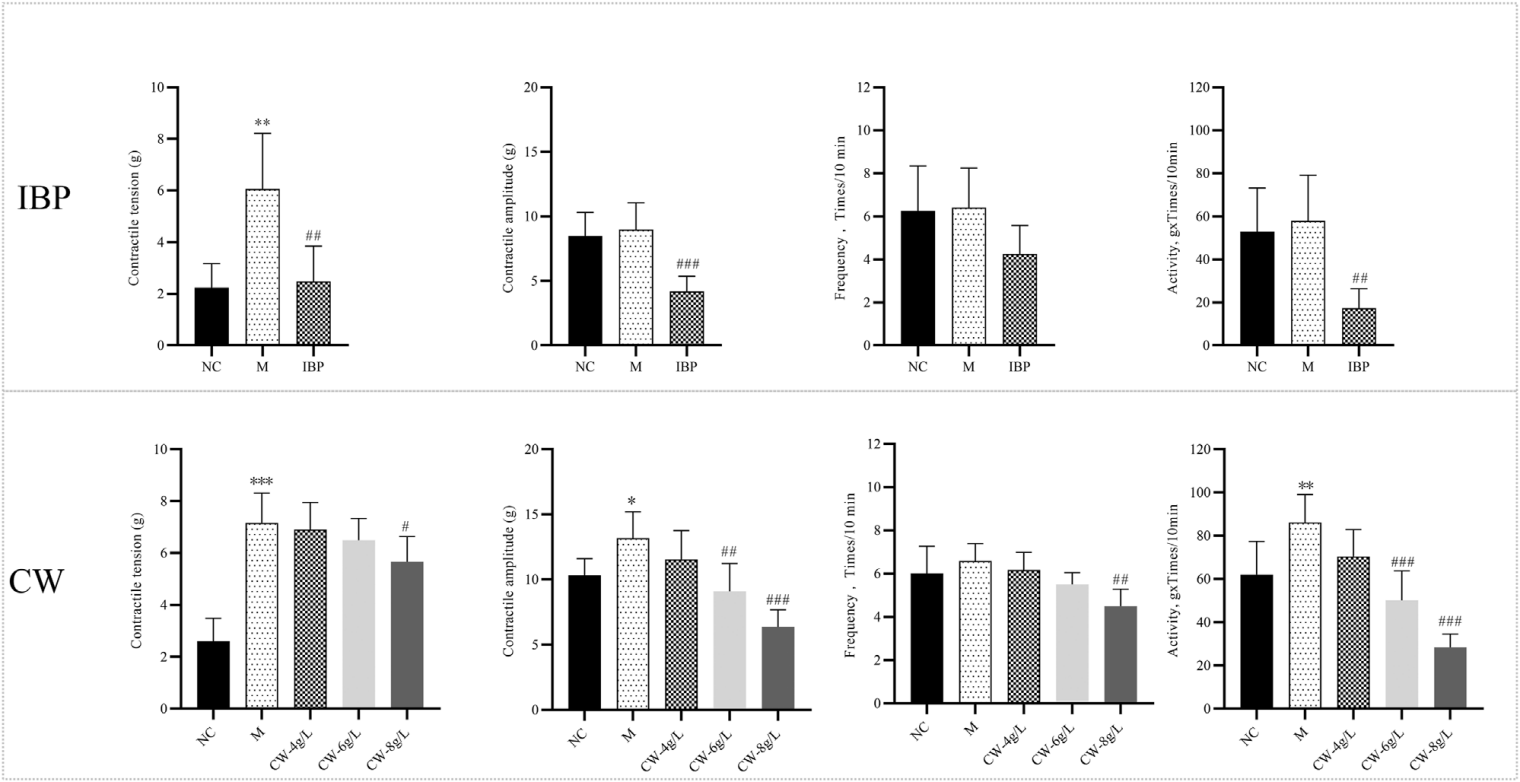
Effect of extract of CW on contractile of the isolated uterus (‾x ± s, *n* = 6). Compared with blank curve, ^*^ *p* < 0.05, ^**^ *p* < 0.01, ^***^ *p* < 0.001; compared with model curve, ^#^ *p* < 0.05, ^##^ *p* < 0.01, ^###^ *p* < 0.001. CW, Curcumae Radix group; IBP, Ibuprofen positive control group.

As shown in Figure 1, compared with the blank curve, the contractile tension and motility of the isolated uterus in the model curve increased significantly (*p* < 0.05*, p* < 0.01*, p* < 0.001), which meant that the uterine smooth muscle had a spasm. Compared with the model curve, the contractile tension, amplitude, and frequency of uterine smooth muscle in the medium to high dose group of CW were inhibited (*p* < 0.05*, p* < 0.01*, p* < 0.001), low dose effect was not significant (*p* > 0.05). The inhibition rates of contraction tension, amplitude, frequency, and motility of the uterus at high doses of CW were 20.95%, 51.59%, 31.61%, and 66.96% in turn, the inhibition rate of CW for spastic contraction of the uterine was close to the inhibition effect of ibuprofen (Supplementary Table S2).

#### 3.1.2 *In vivo* pharmacodynamics: Histopathological examination

**FIGURE 2:**
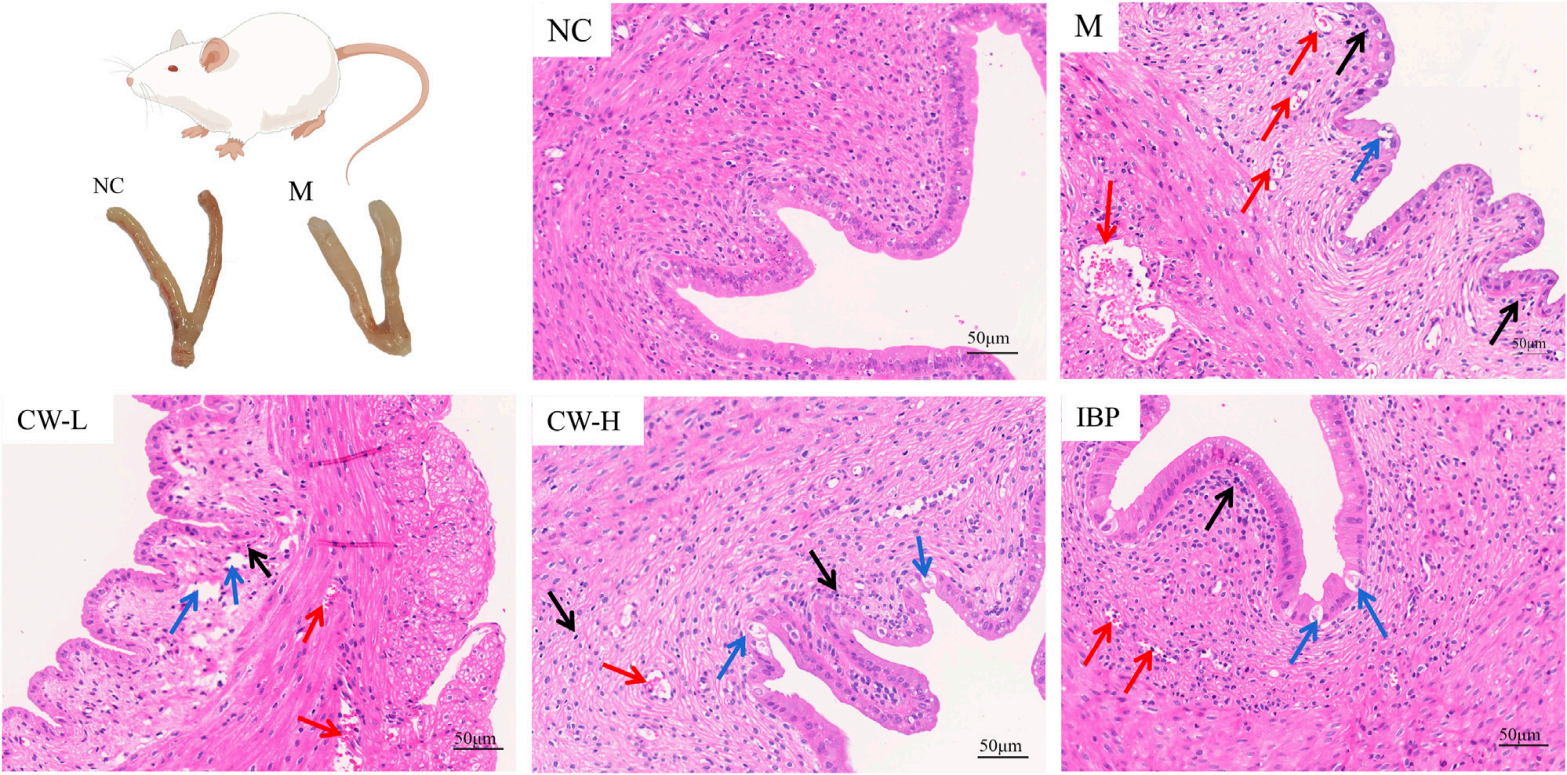
H&E staining of rat uterus tissue (20 × 20). Vacuolated epithelial cells**↑**, inflammatory cells**↑**, and congested cells**↑**. NC, Normal control group; M, Model group; CW-L, CW low-dose group; CW-H, CW high-dose group; IBP, Ibuprofen positive control group.

The histopathological characteristics of the uterus of PD rats were shown in Figure 2. The uterine structure of normal rats in the NC group was clear, including endometrium, myometrium, and adventitia from inside to outside, arranged neatly and tightly. In the M group, the uterine wall was thinner, the structure was disordered, and endometrial epithelial cells were incomplete. The glands in the lamina propria were abnormal and hypertrophic, the endometrium and muscular layer proliferated, and inflammatory cells, such as lymphocytes, were found. A large number of infiltrating inflammatory cells and edema were also observed in the CW-L group. A small amount of inflammatory cell infiltration and edema were observed in the CW-H and IBP groups. Pathological changes in the uterine tissues confirmed the successful establishment of the rat model.

#### 3.1.3 *In vivo* pharmacodynamics: Biochemical assays of PGF_2α_, PGE_2_, β-EP, 5-HT, and Ca^2+^

**FIGURE 3:**
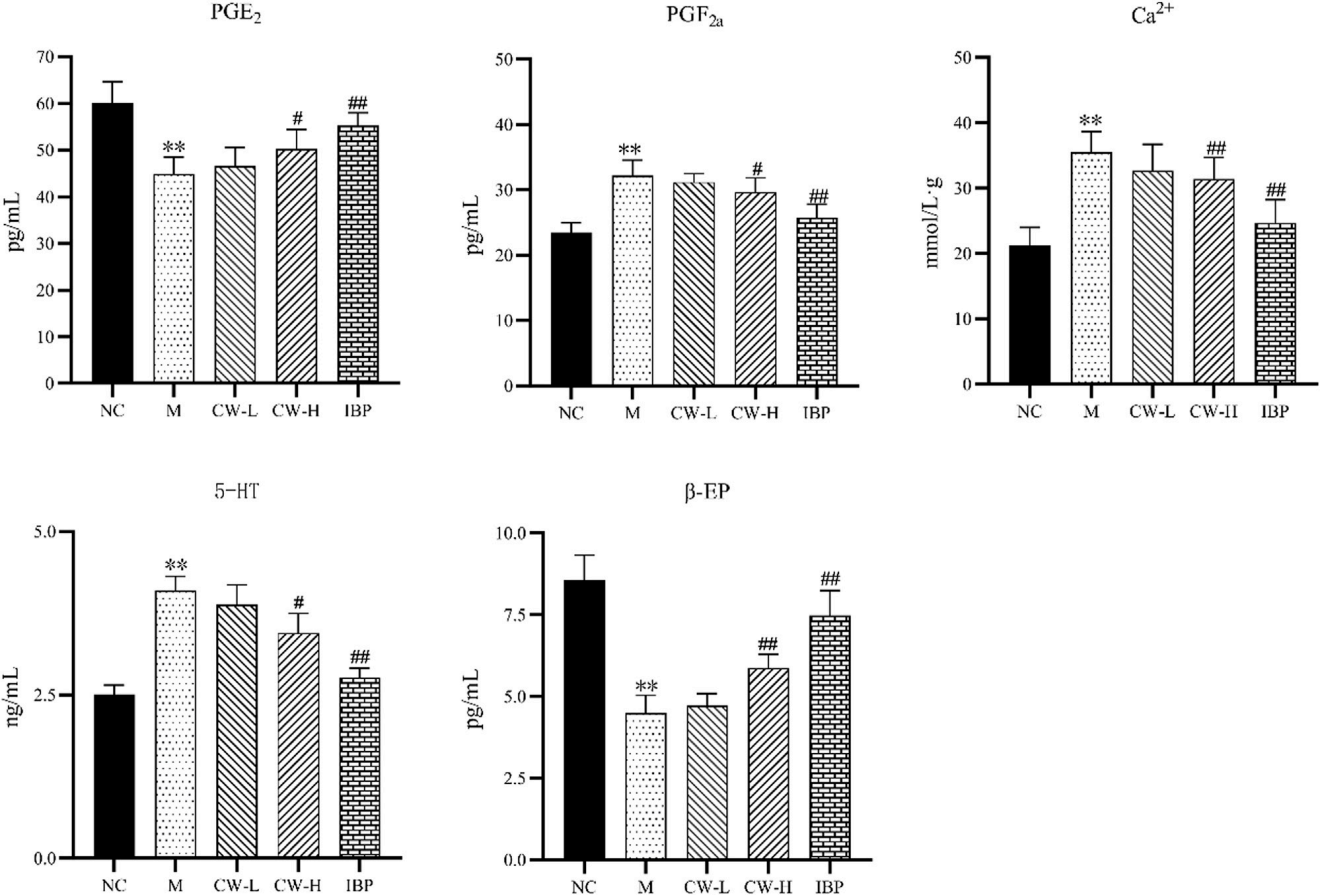
The results of biochemical indexes of rats in each group (‾x ± s, *n* = 8). Compared with the NC group, ^*^ *p* < 0.05, ^**^ *p* < 0.01; compared with the M group, ^#^ *p* < 0.05, ^##^ *p* < 0.01. NC, Normal control group; M, Model group; CW-L, CW low-dose group; CW-H, CW high-dose group; IBP, Ibuprofen positive control group.

As shown in Figure 3, compared with the NC group, PGF_2α_, Ca^2+^, and 5-HT levels were significantly increased, while PGE_2_ and β-EP levels were significantly decreased, in the model group rats (*p* < 0.01) (Tong et al., 2021). Compared with the M group, the modulating effect of the CW-L group was not significant (*p* > 0.05), but both the CW-H and the IBP groups significantly improved each index in the PD rats (*p* < 0.05, *p* < 0.01). This indicates that a certain dose of CW effectively inhibited uterine injury.

### 3.2 Analysis of characterization constituents in drug-containing serum

The total positive and negative ion currents of the drug-containing serum are shown in Supplemental Figure S2A. The drug-containing serum and metabolites of CW were identified and analyzed using Metabolite Pilot 2.0.4 software. A total of 66 components were identified in the drug-containing serum of the PD rats in the CW-H group, including 31 prototype components and 35 metabolites (Supplementary Table S3). As shown in Supplementary Table S3, the metabolic pathways of the sesquiterpenoid components of CW in PD rats mainly included internal hydrolysis, hydrogenation, oxidation, deoxygenation, desaturation, ketone formation, demethylation, desaturation, and carboxylation in one phase. The main metabolic transformation pathways were analyzed using isoprocurcumenol as the representative component (Supplementary Figure S2B).

### 3.3 Predictive results of the mechanism of CW in the treatment of PD

#### 3.3.1 Predictive results of network pharmacology analysis

After the virtual prediction of 66 drug-containing serum components and their metabolites in CW. The predictive results suggest that these core targets (AKT1, AKT serine/threonine kinase 1; PIK3CA, phosphatidylinositol 4,5-bisphosphate 3-kinase catalytic subunit alpha isoform; SRC, proto-oncogene tyrosine-protein kinase; MAPK3, mitogen-activated protein kinase 3, and MAPK1) and signaling pathways (ligand-receptor interaction, calcium signaling pathway, and MAPK signaling pathway) were closely associated with CW for PD. The active ingredients–core targets–critical pathway network was built by Cytoscape 3.9.1 software (Supplementary Figure S3). Topological analysis of the network was performed using the CytoNCA function (Qin et al., 2022). The components were ranked according to the degree value and the top 10 components were curcumadione-M1, curcumenone, curcumenone-M1, 4S-dihydrocurcumenone, 4S-dihydrocurcumenone-M1, linoleic acid, β-elemenone-M1, isoprocurcumenol-M4, procurcumenol, and procurcumenol-M1. The results showed that curcumenone, 4S-Dihydrocurcumenone, procurcumenol, and their metabolites were ranked high in CW, indicating that these components play a major role in the treatment of PD. More details can be found in Supplementary materials S4.

#### 3.3.2 Predictive results of serum non-targeted metabolomics

The One-MAP cloud platform was used to identify the serum samples of each group, and 29 differential metabolites were obtained, which mainly included choline, proline, leucine, arginine, and arachidonic acid (AA). The differential metabolites in the NC and CW-H groups compared to those in the M group are shown in Supplementary Table S4. MetaboAnalyst 5.0 was used to analyze the enrichment of the metabolic pathways of 29 differential metabolites. The differential pathway enrichment plots for the NC and CW-H groups compared with the M group are shown in Supplementary Figure S4A. The results showed that the main metabolic pathway involved in the differential metabolites of the serum samples of each group was AA metabolism, indicating that the metabolic abnormalities in PD rats and the therapeutic effects of CW on PD were closely related to the metabolism of FAs, such as AA.

### 3.4 Verification of the mechanism of CW in the treatment of PD

#### 3.4.1 Regulation of CW on the 5-HTR/Ca^2+^/MAPK signal pathway of PD

**FIGURE 4:**
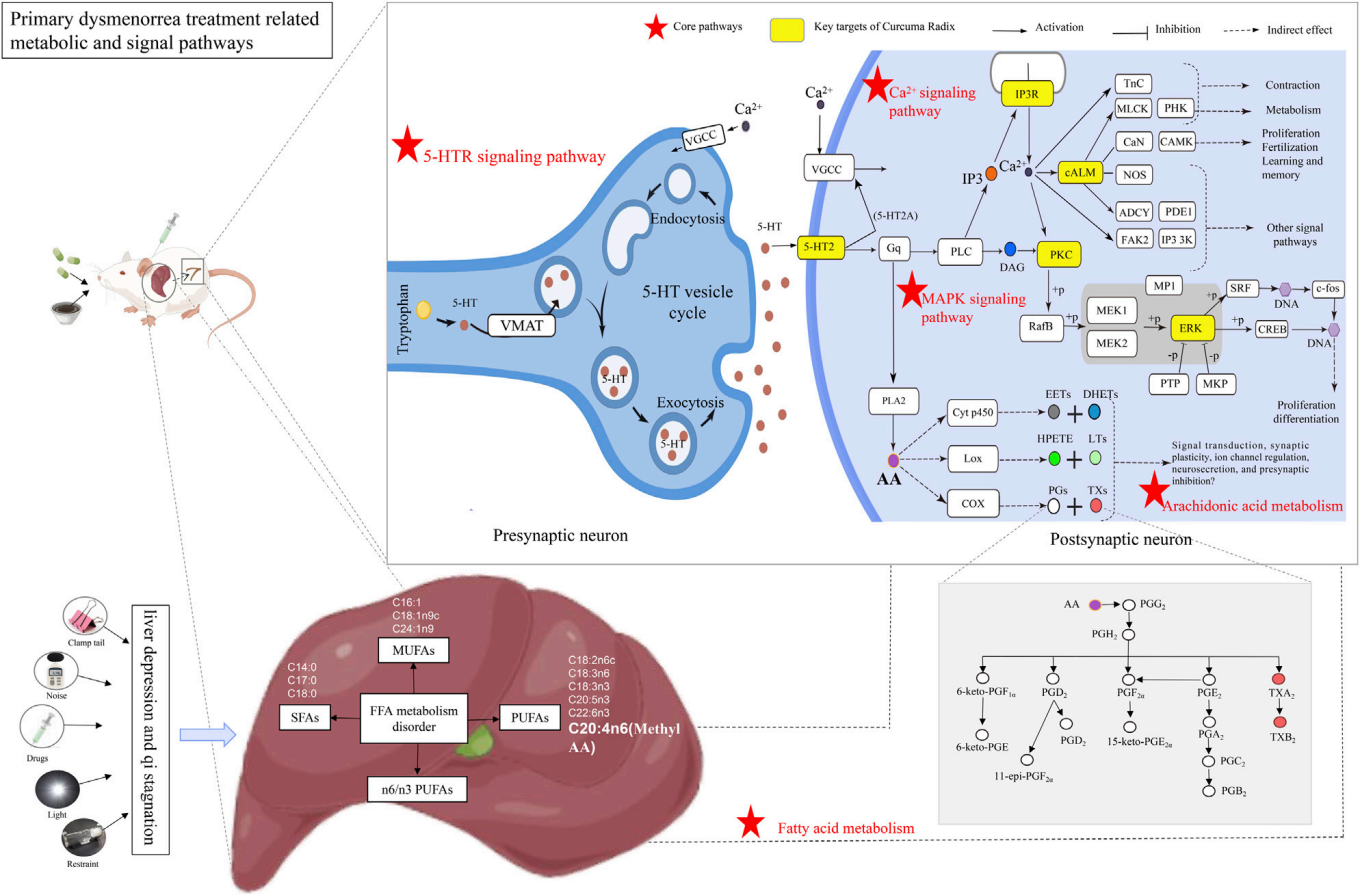
Therapeutic mechanism graph of CW acting on PD. Note: The red pentagram represents the core pathway, the yellow box represents the key target of turmeric action, the arrow indicates the activation effect, the T-shaped arrow indicates the inhibitory effect, and the dotted arrow represents the indirect effect.

Based on the results of network pharmacology prediction, combined with the results of previous studies, the top-ranked 5-HTR/Ca^2+^/MAPK signaling pathway (Figure 4) (Michel et al., 2012; Wu et al., 2021), which may be closely related to PD, was validated by RT-qPCR. At the transcriptional level, the regulatory roles of key genes in this signaling pathway were preliminarily elucidated.

**FIGURE 5:**
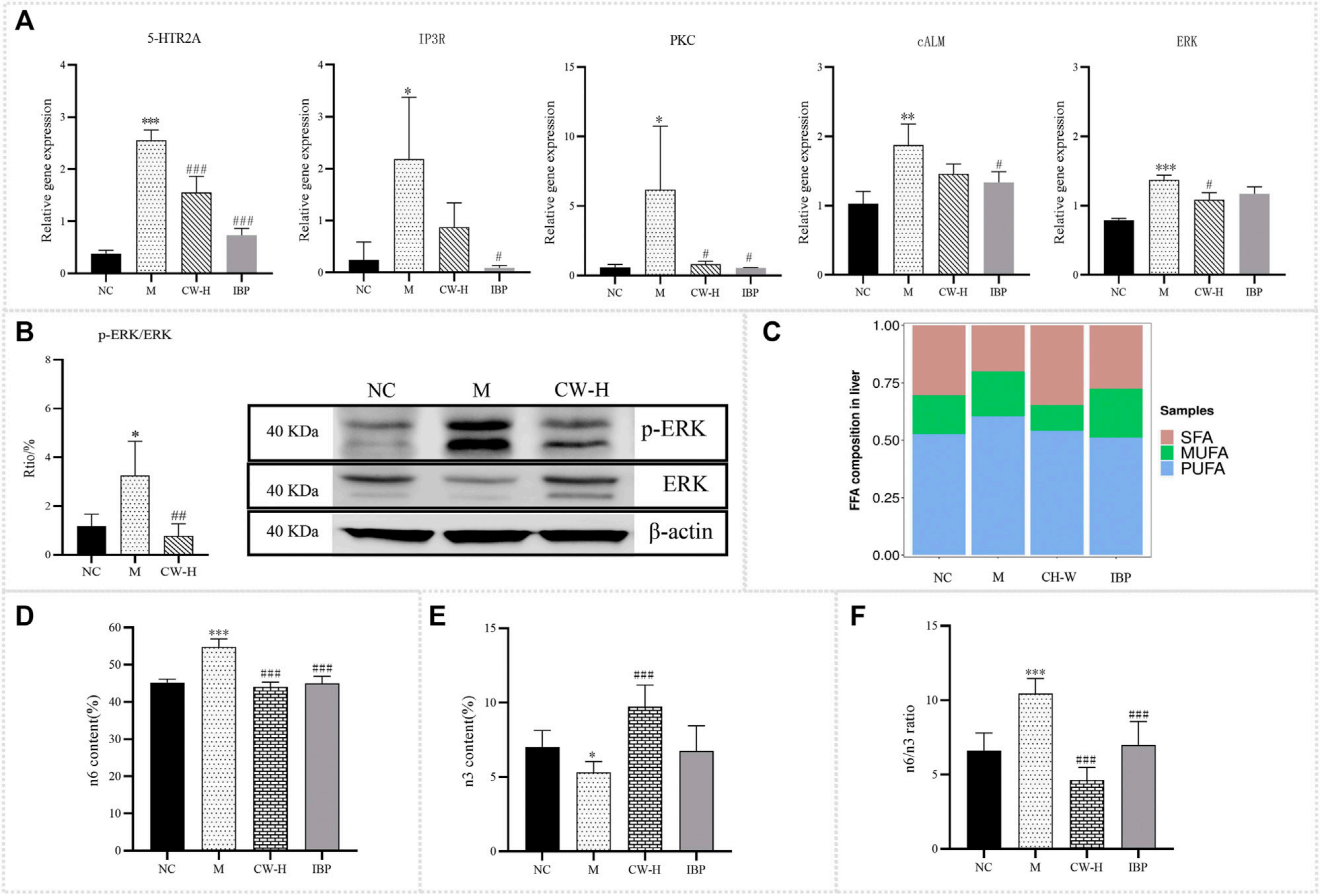
RT-qPCR, WB, and FA metabolomics results of CW against PD. **(A)** Gene expression levels of 5-HTR2A, IP3R, PKC, cALM, and ERK in the uterine tissues of rats in each group (x‾ ± s, *n* = 3). **(B)** Protein expression levels of p-ERK and ERK in the uterine tissues of rats in each group (x‾ ± s, *n* = 3). **(C)** The proportion of SFA, MUFA, and PUFA in liver tissues (x‾ ± s, *n* = 6). **(D)** n6 PUFAs (x‾ ± s, *n* = 6). **(E)** n3 PUFAs (x‾ ± s, *n* = 6). **(F)** n6/n3 ratio (x‾ ± s, *n* = 6). Compared with the NC group, *p < 0.05, **p < 0.01, ***p < 0.001; compared with the M group, ^#^ *p* < 0.05, ^##^ *p* < 0.01, ^###^ *p* < 0.001. NC, Normal control group; M, Model group, CW-H, CW high-dose group; IBP, Ibuprofen positive control group.

RT-qPCR: The gene expression levels of 5-HTR2A, IP3R, PKC, cALM, and ERK in the uterine tissues of the PD rats are shown in Figure 5A. The results showed that the expression of 5-HTR2A, IP3R, PKC, cALM, and ERK genes in the uterine tissues of PD rats was significantly higher than that in the NC group (*p* < 0.05, *p* < 0.01, *p* < 0.001). Compared with the M group, the expression of 5-HTR2A, PKC, and ERK was significantly reduced in the uterine tissue of the CW-H group (*p* < 0.05, *p* < 0.01, *p* < 0.001). The expression of 5-HTR2A, IP3R, PKC, and cALM was significantly reduced in the uterine tissue of the IBP group. The inhibitory effects of CW on the transcriptional levels of key target genes in the 5-HTR/Ca^2+^/MAPK pathway in PD rats were initially verified.

WB: The protein expression levels of p-ERK and ERK in the uterine tissues of the PD rats are shown in Figure 5B, Supplementary Table S5; Supplementary Figure S4. The results showed that the p-ERK/ERK ratio in the uterine tissues of PD rats was significantly higher than that in the NC group (*p* < 0.05). Compared with the M group, the p-ERK/ERK ratio was significantly reduced in the uterine tissue of the CW-H group (*p* < 0.01). This result was consistent with the results of RT-qPCR, and further validates that CW can improve the symptoms of PD by modulating the 5-HTR/Ca^2+^/MAPK signaling pathway.

#### 3.4.2 Regulation of CW on the targeted FA metabolism of PD

Based on the results of serum non-targeted metabolomics prediction and combined with the results of previous studies, AA and other FAs that may be closely related to PD were validated by GC-MS, and the mechanism of CW alleviating PD by regulating FA metabolism (Figure 4) was preliminarily elucidated at the targeted metabolomics level.

Frozen liver samples were taken, and the composition of 36 FAs in liver tissue was determined by GC-MS. The standard curves and stability results for the 36 standards are presented in Supplementary Table S6, S7; Supplementary Figure S5. A total of 16 FAs were detected in this study, while the remaining 20 FAs were not detected (Table 1). The proportions of saturated, monounsaturated, and PUFAs (SFA, MUFA, and PUFA) in the liver are shown in Figure 5C. Compared with the NC group, the proportion of SFAs decreased (30% vs. 20%), whereas the proportion of MUFAs and PUFAs increased (17% vs. 20%; 53% vs. 60%) in the PD group. Compared with the M group, the proportion of SFAs increased, and the proportion of MUFAs and PUFAs decreased in the IBP and CW-H groups. The IBP group showed an increase in the proportion of SFAs (20% vs. 28%) and a decrease in the proportion of PUFAs (51% vs. 60%), with no significant effect on MUFAs (21% vs. 20%).

**TABLE 1:** Effect of CW on the FA content of the liver of model rats (x‾ ± s, *n* = 6, μg/mL).

Groups	NC	M	CW-H	IBP
C14:0	0.0098 ± 0.01	0.0746 ± 0.02^a^	0.0000 ± 0.00^b^	0.0386 ± 0.07
C16:1	0.2419 ± 0.08	0.4303 ± 0.05^c^	0.0499 ± 0.03^d^	0.2656 ± 0.15^d^
C17:0	0.0220 ± 0.01	0.0445 ± 0.01^e^	0.0249 ± 0.00^d^	0.0275 ± 0.01^d^
C18:0	3.2973 ± 0.33	6.0820 ± 0.54^a^	4.8775 ± 0.23^d^	3.9935 ± 0.59^d^
C18:1n9c	1.4502 ± 0.44	5.3087 ± 0.42^a^	1.2222 ± 0.17^d^	2.6261 ± 2.64^b^
C18:2n6c	2.1782 ± 0.42	11.2176 ± 1.60^a^	2.1207 ± 0.38^d^	3.3405 ± 2.24^d^
C18:3n6	0.0052 ± 0.01	0.1567 ± 0.03^a^	0.0051 ± 0.00^d^	0.0080 ± 0.01^d^
C18:3n3	0.0193 ± 0.01	0.2396 ± 0.05^a^	0.0049 ± 0.00^d^	0.0304 ± 0.02^d^
C20:0	0.0033 ± 0.00	0.0040 ± 0.00	0.0022 ± 0.00	0.0071 ± 0.01
C20:1n9	0.0192 ± 0.01	0.0169 ± 0.00	0.0141 ± 0.00	0.0286 ± 0.03
C20:2	0.0535 ± 0.02	0.0634 ± 0.01	0.0397 ± 0.01^f^	0.0593 ± 0.02
C20:3n6	0.0765 ± 0.03	0.0634 ± 0.01	0.0619 ± 0.03	0.0850 ± 0.03
C20:4n6	2.7060 ± 0.23	5.5530 ± 0.64^a^	4.0504 ± 0.31^d^	3.1121 ± 0.11^d^
C20:5n3	0.0325 ± 0.02	0.1568 ± 0.04^a^	0.0096 ± 0.01^d^	0.0314 ± 0.02^d^
C24:1n9	0.1617 ± 0.03	0.2909 ± 0.06^a^	0.3164 ± 0.05	0.2011 ± 0.05^b^
C22:6n3	0.7114 ± 0.11	1.2548 ± 0.27^a^	1.3608 ± 0.20	0.8718 ± 0.18^b^

NC, normal control group; M, model group, CW-H, CW, high-dose group; IBP, ibuprofen positive control group.

Compared with the NC, group. ^a^ *p <* 0.001; compared with the M group. ^b^ *p <* 0.01. ^c^ *p <* 0.01. ^d^ *p <* 0.001. ^e^ *p <* 0.05. ^f^ *p <* 0.05.

SFAs: As shown in Table 1, the C14:0, C17:0, and C18:0 were significantly higher in the M group than in the NC group (*p* < 0.05, *p* < 0.001), whereas C20:0 had no significant effect (*p* > 0.05). Compared with the M group, the composition of C14:0, C17:0, and C18:0 was significantly downregulated in each administration group (*p* < 0.01, *p* < 0.001). Group IBP had no significant effect on C14:0 or C20:0 (*p* > 0.05). The CW-H group had no significant effect on the C20:0 ratio (*p* > 0.05).

MUFAs: As shown in Table 1, the C16:1, C18:1n9c, and C24:1n9 were significantly higher in the M group than in the NC group (*p* < 0.001), whereas C20:1n9 had no significant effect (*p* > 0.05). Compared to the M group, the composition of C16:1 and C18:1n9c was significantly downregulated in each administration group (*p* < 0.01, *p* < 0.001). The IBP group had no significant effect on C20:1n9 (*p* > 0.05). The CW-H group showed no significant effect on C24:1n9 (*p* > 0.05).

PUFAs: As shown in Table 1, the C18:2n6c, C18:3n6, C18:3n3, C20:4n6 (methyl AA), C20:5n3, and C22:6n3 levels were significantly higher in the M group than in the NC group (*p* < 0.001), with no significant effect on C20:2 and C20:3n6 (*p* > 0.05). Compared to the M group, each administration group had significantly lower C18:2n6c, C18:3n6, C18:3n3, C20:4n6, and C20:5n3 levels (*p* < 0.05, *p* < 0.01, *p* < 0.001). The IBP group had no significant effect on C20:2 or C20:3n6 (*p* > 0.05). The CW-H group showed no significant effect on C22:6n3 and C20:3n6 (*p* > 0.05). The PFAs were divided into n6 PUFAs (C18:2n6c, C20:4n6, etc.) and n3 PUFAs (C18:3n3). n3 and n6 PUFAs are antagonistic to each other in various physiological functions to maintain body homeostasis.

As shown in Figures 5D–F, compared with the NC group, n6 PUFA levels were significantly increased (*p* < 0.05, *p* < 0.001), n3 PUFA levels were significantly decreased (*p* < 0.05), and the n6/n3 level was significantly increased in the M group (*p* < 0.001) (Pattanittum et al., 2016). Compared to the M group, n6 PUFA levels were significantly decreased in each administration group (*p* < 0.001), n3 PUFA levels were significantly decreased in the CW-H group (*p* < 0.001), the IBP group had no significant effect on n3 PUFA levels (*p* > 0.05), and the n6/n3 level was significantly decreased in each administration group (*p* < 0.001).

## 4 Discussion

PD is a recurrent spasmodic pelvic pain that occurs in the normal uterus, ovaries, and fallopian tubes during menstruation and is often associated with abnormal mood, heartburn, insomnia, headache, and fainting (Seo et al., 2020). PD, considered a major public health problem, is one of the most common gynecological conditions (Libarle et al., 2018). PD negatively affects the daily life and work of many patients because the treatment of PD with NSAIDs has not yet reached its optimal state (Ferries-Rowe et al., 2020). In contrast, in TCM, it is believed that PD is related to the liver, and herbal medicines that dredge the liver and invigorate blood are commonly used to treat PD, such as CW. However, the material basis and mechanism of CW for PD have not been systematically studied.

In the present study, a widely used isolated uterine model *in vitro* and a PD model *in vivo* were established, and the symptoms closely resembled the clinical manifestations of human PD (Tong et al., 2021). In the construction of the animal model, SD female rats are more suitable for the PD model because of their lower cost, short reproductive cycle, and sensitivity to sex hormones; Estradiol benzoate is an estrogenic drug that synchronizes the uterus and enhances uterine sensitivity; OT can induce uterine spastic contraction and produce pain to simulate PD symptoms (Kidd et al., 2015); Combined stimuli (sound, light, tail pinch, restraint, etc.) can induce undesirable emotions such as restlessness and anger in rats; Epinephrine hydrochloride simulates mania, and enhances the stress response of rats, creating a state of Qi stagnation. The use of estradiol benzoate and OT supplemented with other means of restriction has been reported to better mimic the symptoms of PD, and the aforementioned method is currently the most common method of PD model with TCM characteristics (Tong et al., 2021). However, because of the absence of menstruation in rats and the complex pathogenesis of PD, the experimental PD model needs further improvement.

In the pharmacological evaluation, most of the results in the M group were significantly different compared to the NC group and were consistent with many clinical studies, reported in the literature, implying successful PD model construction (Sharghi et al., 2019; Zhang et al., 2021; Hong et al., 2022). *In vitro* pharmacodynamic experiments, the results showed that CW significantly inhibited the abnormal contractile tone, amplitude, frequency, and activity of the uterine smooth muscle in a dose-dependent manner (*p* < 0.05, *p* < 0.01, *p* < 0.001). To further investigate the mechanism of CW in the treatment of PD, *in vivo* pharmacodynamic experiments were continued and also corroborated the results of *in vitro* pharmacodynamic experiments. Pathological tissue sections and biochemical factors such as PGE_2_ are commonly used to assess PD progression (Sharghi et al., 2019; Zhang et al., 2021; Arabnezhad et al., 2022; Hong et al., 2022). Histopathological changes in the uteri of PD rats usually include uterine wall thinning, structural disorders, glandular abnormalities, marked hyperplasia, and inflammatory cell infiltration (Tong et al., 2021). PGE_2_ can effectively inhibit the spontaneous activity of uterine smooth muscle, while PGF_2α_ can promote the contraction of uterine smooth muscle and reduce the amount of uterine blood perfusion. If the level of PGE_2_ decreases and that of PGF_2α_ increases in the body, it can lead to excessive contraction of the uterus and produce spasmodic contractions, which, in turn, induce PD (Zhang et al., 2021). β-EP regulates female reproductive neurotransmitters and maintains a stable neuroendocrine environment. If the level of β-EP decreases during the luteinizing hormone phase in women, it leads to a decrease in the regulation of the uterus and subsequently induces PD (Zhang et al., 2021). Another study showed that 5-HT in the peripheral environment is an inflammatory factor that mediates pain, while peripheral blood 5-HT and estrogen levels are positively correlated. When PD occurs, blood levels of 5-HT are elevated (Viguier et al., 2013). Ca^2+^ is a key factor that regulates the contraction of uterine smooth muscle cells. When the intracellular Ca^2+^ concentration in uterine smooth muscle cells exceeds 10^–6^ M, it induces uterine contraction. When the inward flow of extracellular Ca^2+^ leads to an increase in intracellular Ca^2+^ levels, it will, in turn, lead to the contraction of blood vessels and uterine myometrium and insufficient blood supply to the endometrium, which then induces PD (Sharghi et al., 2019). Here, CW significantly ameliorated the symptoms of PD, as evidenced by changes in the pathological state of the uterus (Figure 2) and biochemical factors such as PGE_2_ (Figure 3).

In a classical spectral study, 66 metabolites in drug-containing serum were identified by UPLC-Q-TOF-MS technology, of which 31 were prototype components. Most of these compounds were sesquiterpenes, indicating that these may be the core substances participating in the treatment of PD. In addition, it was found that although curcumin components had significant blood-activating activity, this class of components did not reach the detection limit in drug-containing serum. This result is consistent with reports of its low oral bioavailability, which may be related to the poor solubility of curcumin components in gastrointestinal fluids or their rapid degradation at intestinal pH (Zhu et al., 2022). These results are similar to those reported in previous studies (Tong et al., 2021; Fei et al., 2022). To understand the process of action of these components in PD rats, the above components were predicted using network pharmacology. The predicted results suggest that CW anti-PD is associated with AKT1, PIK3CA, SRC, MAPK3, MAPK1, and other targets. Curcumenone, procurcumenol, and their metabolites in CW may treat PD by regulating serotonergic synapses, Ca^2+^ signaling pathway, MAPK signaling pathway, and PI3K/AKT signaling pathway (Figure 4). Ca^2+^ signaling pathway and MAPK signaling pathway reported to be associated with improvement of PD (Tong et al., 2021); PI3K/AKT pathway is associated with improved endometrial hyperplasia (Liu et al., 2023). 29 differential metabolites in drug-containing serum were identified by the One-MAP cloud platform, which mainly included arginine, AA, and so on (Supplementary Table S4). NO is produced by arginine under the action of nitric oxide synthase, relaxes uterine smooth muscle cells, dilates blood vessels, and has an analgesic effect. AA mediates the production of inflammatory mediators through COX-2 catalysis, which is a synthetic substrate of PGF_2α_ and PGE_2_, induces platelet aggregation, and regulates smooth muscle contraction and the production of inflammatory cytokines (Zhang et al., 2021; Hong et al., 2022).

Based on network pharmacology predictions, RT-qPCR and WB were used to verify the key targets for the regulation of the 5-HTR/Ca^2+^/MAPK signaling pathway at the level of gene and protein expression (Figure 5). The results showed that CW downregulated the gene expression of 5-HTR2A, IP3R, PKC, cALM, and ERK, and downregulated the protein expression of p-ERK/ERK in PD rats, which were consistent with relevant literature reports (Peng et al., 2020; Huang et al., 2021; Tong et al., 2021). The inhibitory effect of CW on the transcriptional and protein levels of key targets in the 5-HTR/Ca^2+^/MAPK pathway in PD rats was verified. This pathway may affect the occurrence and development of PD by regulating emotion, pain, endocrine function, smooth muscle contraction, and inflammation. As shown in Figure 5, 5-HT acts on related receptors, such as 5-HT2 and activated phospholipase C, to produce IP3, which leads to PD by acting on IP3R, increasing in the intracellular Ca^2+^ concentration. The MAPK signal pathway can be activated by Ca^2+^, resulting in high levels of MAPK and ERK, and the expression of some inflammatory factors increased. Studies have shown that when Ca^2+^ enters a large number of cells, uterine smooth muscle contracture is caused by overloaded Ca^2+^, leading to continuous vasoconstriction, and phospholipase A2 (phospholipaseA2, PLA2) and COX are activated, which regulate AA and other FA metabolism, producing PGs and other factors, and finally leading to vasoconstriction and inflammatory reactions, causing PD. To sum up, PD has been considered to be highly associated with inflammation. Inflammation is closely linked to protein phosphatases, such that the balance between protein phosphorylation/dephosphorylation is regulated by aberrant expression or disruption of the imbalanced activity of phosphatases and protein kinases (Pan et al., 2022). This report is consistent with the WB results in this study.

Based on serum non-targeted metabonomics predictions, targeted metabolomics was used to verify the downstream metabolic pathways that regulate the 5-HTR/Ca^2+^/MAPK signaling pathway (Figure 4). The results showed that CW relieved the symptoms of PD by regulating FA metabolism in PD rats. It was preliminarily verified that CW might play a role in the treatment of PD by improving FA metabolism. It has been reported that n3 PUFAs can inhibit inflammation, regulate blood lipids, and protect the cardiovascular system in various ways. n6 PUFAs can promote immune responses and enhance body resistance; however, they can also promote inflammation. When the proportion of n6/n3 increased, the proliferation and metastasis of tumor cells were mediated, inflammation and thrombosis were promoted, and the concentration of C20:4n6 and PGs in the ovaries of female rats was significantly increased. These substances were also closely related to PD, indicating that the ratio of n6/n3 was related to PD (Yi et al., 2012; Zhang et al., 2021; Hong et al., 2022). CW significantly decreased the n6/n3 ratio in the liver (*p* < 0.001), indicating that it was able to alleviate the symptoms of PD by improving the FA metabolic pathway (Figure 5).

## 5 Conclusion

Given the unclear mechanism of CW in the treatment of PD, *in vitro/vivo* pharmacodynamics, metabonomics was used in combination with transcriptomics, UPLC-Q-TOF-MS/MS, GC-MS, RT-qPCR, and WB were used in this study to initially explained the regulatory effect of CW on the 5-HTR/Ca^2+^/MAPK signaling pathway, which indirectly affects FA metabolism, thereby improving uterine spasm, the inflammatory reaction, and AA metabolism. It was shown that CW had multi-component, multi-pathway, and multi-target characteristics in the treatment of PD. This study provides a basis for the subsequent development and utilization of CW. Later, the effective substances and mechanisms of CW for relieving PD will be verified by a molecular interaction analyzer. Further experiments will be conducted to investigate the association between 5-HTR/Ca^2+^/MAPK signaling pathways and FA metabolism and their regulation of PD.

## Data Availability

The original contributions presented in the study are included in the article/Supplementary Material, further inquiries can be directed to the corresponding authors.

## Abbreviations

CW, *Curcumae* Radix (*Curcuma wenyujin*); PD, Primary dysmenorrea; TCM, Traditional Chinese medicine; OT, Oxytocin; PG, Prostaglandin; FA, Fatty acid; NSAIDs, Non-steroidal anti-inflammatory drugs; NC group, Normal control group; M group, Model group; CW-L group, CW low-dose group; CW-H group, CW high-dose group; IBP group, Ibuprofen positive control group; QC, Quality control; Ca^2+^, Calciumion; 5-HTR2A, 5-Hydroxytryptamine receptor 2A; IP3R, Inositol 1,4,5-trisphosphate receptor; GC-MS, as Chromatography-Mass Spectrometer; *t-test,* Student’s t-test; AA, Arachidonic acid; SFA, Saturated fatty acid; MUFA, Monounsaturated fatty acid; PUFA, Polyunsaturated fatty acid; PGE_2_, Prostaglandin E_2_; PGF_2α_, Prostaglandin F_2α;_ β-EP, β-Endorphin; 5-HT, Serotonin; UPLC-Q-TOF-MS/MS, Ultra-high performance liquid chromatography coupled with quadrupole time-of-flight tandem mass spectrometry; RT-qPCR, Real-time fluorescence quantitative polymerase chain reaction; WB, Western blotting.
