# Supplementary material for: The therapeutic mechanism of Curcumae Radix against primary dysmenorrea based on 5-HTR/Ca2+/MAPK and fatty acids metabolomics
Source: Front Pharmacol. 2023 Mar 9;14:1087654. doi: 10.3389/fphar.2023.1087654 (PMC10034069; doi:10.3389/fphar.2023.1087654)
Supplement: Supplementary file 1 [file DataSheet1.zip › Supplemental materials/Supplemental files S1.docx]

CW slices were prepared as follows: The CW was infiltrated overnight, and then cut into 2  mm thickness, dried at 40 ℃. CW extractive liquid was prepared as follows: CW slices were refluxed for 1.5 h with 90% ethanol 15 times and extracted twice. The residues were extracted for 1.5 h with water 10 times. The above extractive liquid of CW was combined and filtered through a four-layer mesh. The mixed solution was vacuum-concentrated by a rotary evaporator at 50 ℃. Finally, CW extractive liquid containing 0.2 g·mL^-1^ of raw medicine was obtained for subsequent experiments.

The maintenance feed for experimental rats and experimental shavings cushion material were got from Jiangsu Cooperative Pharmaceutical and Biological Engineering Co., Ltd. (Jiangsu, China). Epinephrine Hydrochloride injection (Lot: 62009111, 1 mL: 1 g) purchased from Suicheng Pharmaceutical Co., Ltd. Estradiol benzoate injection (Lot: 20210302, 2 mL: 4g) purchased from Hefei Zhonglong Shenli Animal Pharmaceutical Co., Ltd. (Anhui, China). Oxytocin injections (Lot: 201221-1, 1 mL: 10 U) were purchased from Maanshan Fengyuan Pharmaceutical Co., Ltd. (Anhui, China). Ibuprofen tablets (Lot: 2105221) were purchased from Shandong Fangming Pharmaceutical Group Co., Ltd. (Shandong, China). Pentobarbital sodium (China Pharmaceutical (Group) Shanghai Chemical Reagent Co., Ltd., batch number: F20020405). 4 % paraformaldehyde was purchased from Beijing Lanjie Technology Co., Ltd. Prostaglandin E_2_ (PGE_2_) kit (Lot: Apr 2022), prostaglandin F_2α_ (PGF_2α_) kit (Lot: Apr 2022), β-endorphin (β-EP) kit (Lot: Apr 2022), serotonin (5-HT) kit (Lot: Apr 2022), and calcium ion (Ca^2+^) test kit (Lot: Apr 2022) were purchased from Ruixin Biotechnology Co., Ltd.

Authentic standards of furanodiene (Lot: 111824-201102), β-elemene (Lot: 100268 - 201903), and demethoxycurcumin (Lot: 112003-201501) were obtained from National Institute for food and drug control (Beijing, China). Curzerene (Lot: M20N6K5901), germacrone (Lot: J27J9T66646), curcumenol (Lot: R21M9F61833), isocurcumenol (Lot: Z14J10X90606) were purchased from Yuanye Biotechnology Co., Ltd (Shanghai, China). Curdione (Lot: lw17090612), curcumin (Lot: lw17091410), bisdemethoxycurcumin (Lot: lw16090905) were bought from Liangwei Biotechnology Co., Ltd (Nanjing, China). Furanodienon (Lot: 7883) was got from Shidande Standard Technical Service Co., Ltd (Shanghai, China). The purities of them were all ≥ 98%. 37 kinds of FA methyl esters mixed standard (Lot: 65540060) were purchased from Shanghai Anpu Experimental Technology Co., Ltd. TRIzol™ Reagent (15596018) was purchased from Semel Fisher Technology Co., Ltd. HyPure TMMolecular Biology Grade Water (SH30538.02) was purchased from HyClone. HiScript II 1st Strand cDNA Synthesis Kit (R211-01) and AceQ qPCR SYBR Green Master Mix (Low ROX Premixed) (Q131-02) were purchased from Vazyme.

In this study, the acetonitrile (LC-MS grade, lot: l0901229730) and formic acid (UHPLC grade, lot: 75C1812LP) which was used for the UHPLC analysis were purchased from Merck Co. Inc. (Germany). The methanol (LC-MS grade, lot: H4870288) used for the elution of extracting solution was purchased from Anpel (Shanghai, China). The methanol (HPLC-grade, lot: 20210104) used for the analytical samples’ pretreatment was purchased from Yonghua Chemical Co., Ltd (Suzhou, China). The ultra-pure water was obtained from a Milli‑Q system (Millipore Corporation, Bedford, MA, USA). SPE columns (LC-C_18_, 500 mg/mL) were purchased from ANPLE Scientific Instrument (Shanghai, China). Xylene (Lot: 10023418）and neutral gum (Lot: 10004160) were purchased from National Pharmaceutical Group Chemical Reagent Co., Ltd. HE dye solution Lot (Lot: G1005), differentiation solution (Lot: G1005-3) and blue return solution (Lot： G1005-4) were all purchased from servicebio. Other chemicals used in this study were analytical reagent grade.
