# Supplementary material for: The therapeutic mechanism of Curcumae Radix against primary dysmenorrea based on 5-HTR/Ca2+/MAPK and fatty acids metabolomics
Source: Front Pharmacol. 2023 Mar 9;14:1087654. doi: 10.3389/fphar.2023.1087654 (PMC10034069; doi:10.3389/fphar.2023.1087654)
Supplement: Supplementary file 1 [file DataSheet1.zip › Supplemental materials/Supplemental files S2.docx]

With a Shimadzu UPLC system (Japan), coupled with a DGU-20A5R online solvent degasser, LC-30AD binary liquid pump, SIL-30SD autosampler, CTO-30A column incubator, AB Triple TOFTM 5600+ system equipped with an electrospray ion (ESI) source (AB SCIEX, CA) and an Agilent Zorbax SB-C18 (2.1 mm × 100 mm, 1.8 µm) column, the chromatographic and mass spectrometric conditions established by the group previously were used to perform the assay (Qin et al., 2022).

In this study, the instrument was operated in positive (ESI^+^) and negative ion modes (ESI^−^) under conditions established in previous articles (Fei et al., 2022; Qin et al., 2022). Raw data were obtained using the Analyst ®TF 1.6 software (AB Sciex Corporation, USA). The drug metabolites in the serum were searched for using the MetabolitePilot 2.0.4 software (AB SCIEX, USA). The raw data were acquired using the Analyst TF 1.6 software. To track the serum compositions of CW and identify the metabolic pathways of the drug *in vivo*. The Metabolite Pilot 2.0.4 software (AB SCIEX, USA) metabolite analysis software was used to identify the components in the drug-containing serum (Fei et al., 2022).
