# Supplementary material for: The therapeutic mechanism of Curcumae Radix against primary dysmenorrea based on 5-HTR/Ca2+/MAPK and fatty acids metabolomics
Source: Front Pharmacol. 2023 Mar 9;14:1087654. doi: 10.3389/fphar.2023.1087654 (PMC10034069; doi:10.3389/fphar.2023.1087654)
Supplement: Supplementary file 1 [file DataSheet1.zip › Supplemental materials/Supplemental files S3.docx]

**Regulation of CW on the 5-HTR/Ca^2+^/MAPK signal pathway of PD**

**RT-qPCR:** Total uterine tissue cellular RNA was extracted and reverse transcribed according to the RNA extraction and reverse transcription kits, respectively. Specific primers were designed based on previous studies (**Supplemental material** **Table. S1**). β-Actin was used as an internal reference gene, and the following reaction system was prepared using the AceQ qPCR SYBR Green Master Mix reagent kit: 2 × qPCR Mix (10 μL), 10 μM gene primer (2.0 μL), reverse transcription product (20 μL), ddH2O (6.0 μL), and ROX1 (0.4 μL). RT-qPCR was performed using Applied Biosystems QuantStudio 6-Flex (Thermo Fisher, USA) instruments according to the following procedure: predenaturation was performed at 95 ℃ for 10 min; the 95 ℃, 15 s → 60 ℃, 60 s process was repeated 40 times; and finally, the temperature was increased from 75 ℃ to 95 ℃ at a rate of 1 ℃ per 20 s. The relative expression levels of the factors were calculated using the 2^−ΔΔCT^ method, and each gene was analyzed in triplicate (Wu et al., 2019; Wang et al., 2017).

**WB:** The total protein of the uterine tissues was first extracted by KZ-III-F tissue homogeniser (Servicebio, China), the protein solution was added to 5 reduced protein loading buffers at a ratio of 4:1, denatured in a boiling water bath and then subjected to SDS-PAGE electrophoresis, membrane transfer, immunoassay and chemiluminescence experiments in sequence. The data were analyzed using alphaEaseFC grey-scale analysis software (Alpha Innotech, USA) and Adobe PhotoShop image analysis software (Adobe, USA).

**Regulation of CW on targeted FA metabolism of PD**

**Sample preparation method:** Samples were weighed in 50 mg into a 2 mL grinding tube accurately, and grinding beads and 1 mL methylene chloride: methanol (v/v = 1:1) were added. Then, the mixture was ground in a freezing grinder (50 Hz) for 3 min. Sonicated at low temperature for 15 min, let stand at -20 ℃ for 15 min. Centrifugation at 4 ℃, 13000 rpm for 10 min, the supernatant was pipetted 500 μL into a 1.5 mL EP tube, blow dried by nitrogen, and 0.5 mL of methylation reagent (0.5 mol/L sodium hydroxide methanol solution) was added, vortexed for 30 s and bathed in water at 60 ℃ for 0.5 h. After it cooled down, 0.5 mL of hexane was added, swirled around for 30 s, centrifugation at 4 ℃, 13000 rcf for 10 min, 100 μL of the upper layer (n-hexane layer) solution was taken into the injection vial for GC-MS detection.

**Preparation of medium and long chain FA methyl ester standard solution**: 100 mg of 36 FA methyl esters mixed standard was dissolved by 1 mL of dichloromethane to obtain 36 FA methyl esters mixed standard stock solution A. 100 μL of A solution was added to 900 μL of dichloromethane to obtain Intermediate B, a mixed standard of 36 FA methyl esters, which was diluted and detected by 8890 - 7000 D GC-MS (Agilent Technologies Inc. CA, UAS). In this study, mixed standard solutions at a concentration of 10 μg/mL were used as quality control (QC) samples with one shot of QC sample in every 5-10 samples, mainly for assessing the stability of the analytical system.

**Liquid chromatographic and mass spectrometric conditions**

**Chromatographic condition:** Agilent DB-FastFAME capillary column (20m, 0.18mm, 0.2μm, Agilent J&W Scientific, Folsom, CA, USA). High-purity helium was used as a carrier gas (purity not less than 99.999%), the flow rate was 1.0 mL/min, and the temperature of the injection port was 230℃. The injection volume was 1 μL, fractional injection (fractional ratio 50:1), and the solvent delay was 1.0 min. The programmed temperature rise was set as follows: The initial temperature of the column was 80℃, kept for 0.5 min, increased at 70 °C/min to 175 °C, then increased at 8 °C/min to 230°C, post-run time 2 min at 80 ℃.

**MS conditions:** Electron bombardment ion source (EI), Ion source temperature 230 ℃, four-stage rod temperature 150 ℃. The transmission line temperature was 240 °C, and the electron energy was 70 eV. The scanning mode was selected ion scanning mode (SIM).
