# Supplementary material for: The therapeutic mechanism of Curcumae Radix against primary dysmenorrea based on 5-HTR/Ca2+/MAPK and fatty acids metabolomics
Source: Front Pharmacol. 2023 Mar 9;14:1087654. doi: 10.3389/fphar.2023.1087654 (PMC10034069; doi:10.3389/fphar.2023.1087654)
Supplement: Supplementary file 1 [file DataSheet1.zip › Supplemental materials/Supplemental files S4.docx]

After the virtual prediction of ADMET of 66 drug-containing serum components and their metabolites in CW, 55 active ingredients were identified as eligible for screening. The Swiss Target Prediction and Drug Bank databases were used to search for targets of the above active ingredients, and a total of 503 active ingredient targets were obtained after the deletion of duplicate entries. By searching the Gene Cards, TCMSP, HERB, CTD, and DisGeNET databases for targets related to PD, 15,035 targets were collected after deleting duplicate entries (Qin et al., 2022).

The 503 targets corresponding to the active ingredients of CW were intersected with the 15,035 disease targets related to PD to obtain 413 common targets, which were the potential targets of CW for the treatment of PD. These targets were uploaded to the STRING database and the highest confidence level (0.9) was set (Qin et al., 2022). The TSV data file of PPI was downloaded, and imported into Cytoscape 3.9.1 software, and the CytoNCA tool was used for topological analysis, with the node size reflecting the degree value size and the node color shade representing the target importance (Qin et al., 2022). A total of 73 core targets with degree values > 8 (The degree value should be greater than 2 times the median) were screened, and the top five targets were AKT serine/threonine kinase 1 (AKT1), phosphatidylinositol 4,5-bisphosphate 3-kinase catalytic subunit alpha isoform (PIK3CA), proto-oncogene tyrosine-protein kinase (SRC), mitogen-activated protein kinase (MAPK) 3 and MAPK1 (**supplemental materials** **Fig. S3A)**.

The DAVID database was used for GO functional enrichment analysis of potential targets and KEGG signaling pathway enrichment, and "*Homo sapiens*" was selected as the species and background (Qin et al., 2022). A total of 477 entries for biological processes, 66 entries for cell composition, and 160 entries for molecular functions were obtained (*p* < 0.01). The top 10 entries were selected to draw a bar chart, see (**supplemental materials** **Fig. S3B)**. Meanwhile, 173 enrichment results were obtained for KEGG signaling pathways (*p* < 0.01), from which 30 pathways with proven association with PD were screened by p-value, including neuroactive ligand-receptor interaction, calcium signaling pathway, MAPK signaling pathway, and others (**supplemental materials** **Fig. S3C)**.

The core targets and their chemical components were imported into Cytoscape 3.9.1 software (Qin et al., 2022), and the top 30 signaling pathways and their related targets were integrated to build the *active ingredients–core targets–critical pathway* network (**supplemental materials** **Fig. S3D**).
