# Supplementary material for: The therapeutic mechanism of Curcumae Radix against primary dysmenorrea based on 5-HTR/Ca2+/MAPK and fatty acids metabolomics
Source: Front Pharmacol. 2023 Mar 9;14:1087654. doi: 10.3389/fphar.2023.1087654 (PMC10034069; doi:10.3389/fphar.2023.1087654)
Supplement: Supplementary file 1 [file DataSheet1.zip › Supplemental materials/Supplemental materials-figureS1-S5.docx]

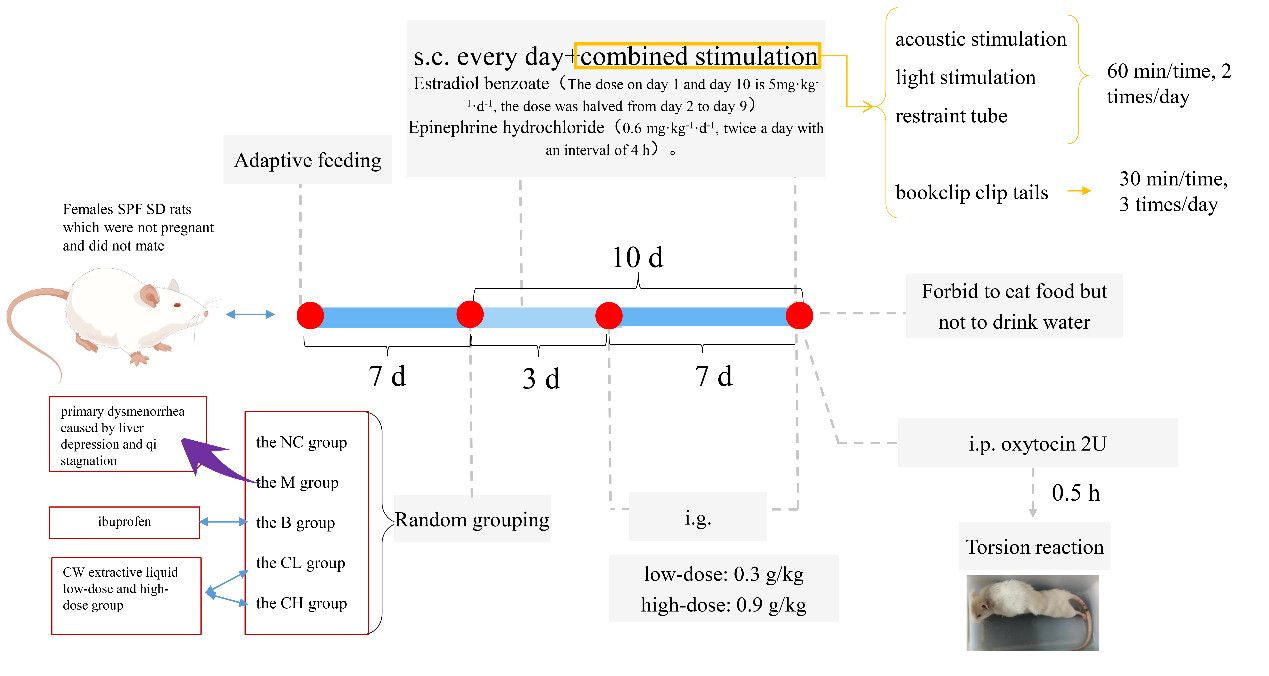


**Fig. S1** Flow chart of experimental animal modeling


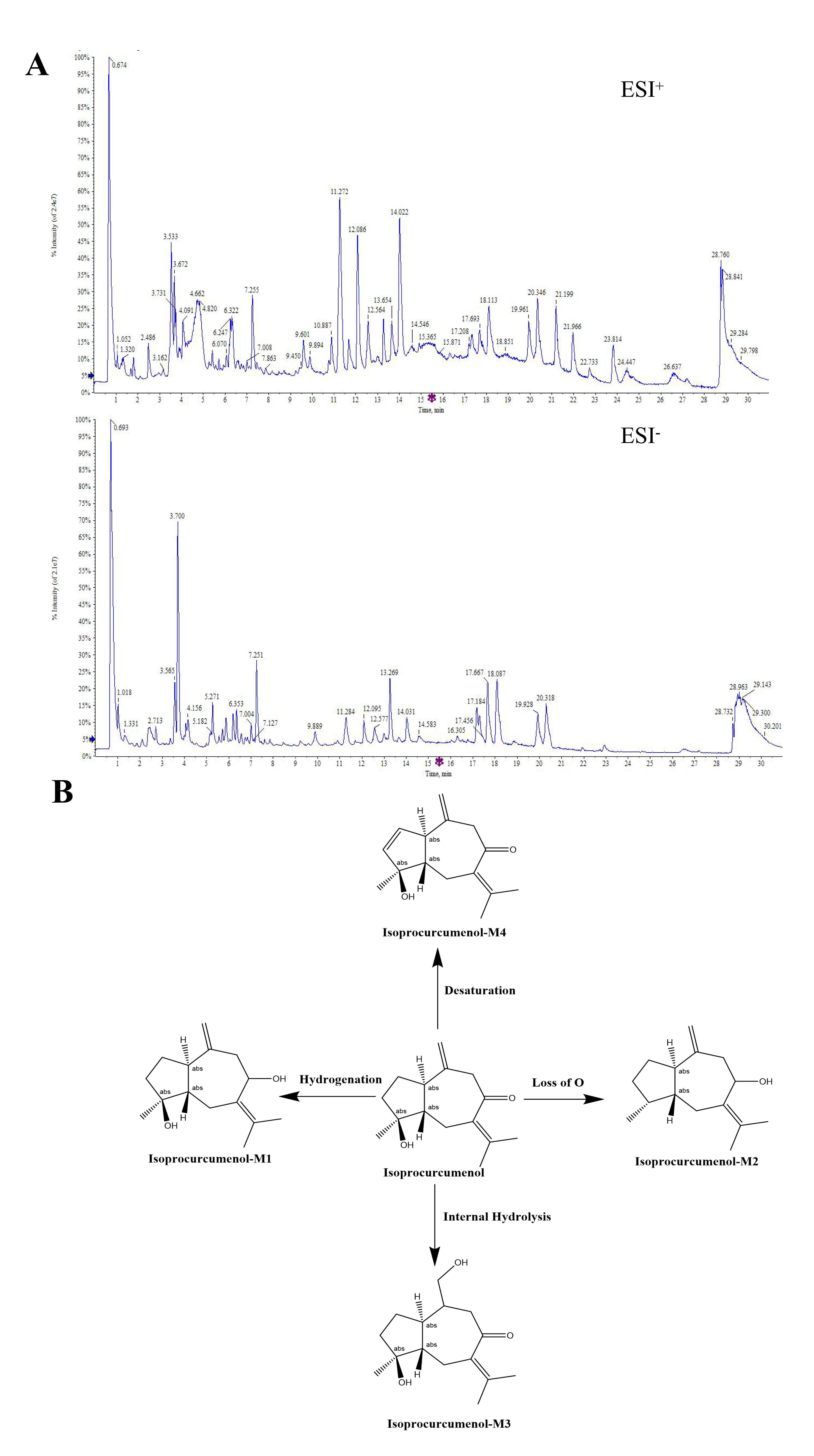


**Fig. S2** Mass Spectrometry Analysis of Serum Containing Drugs. (A) Typical TIC diagram of drug-containing serum; (B) Inferred metabolic pathway of isoprocurcumenol in serum.


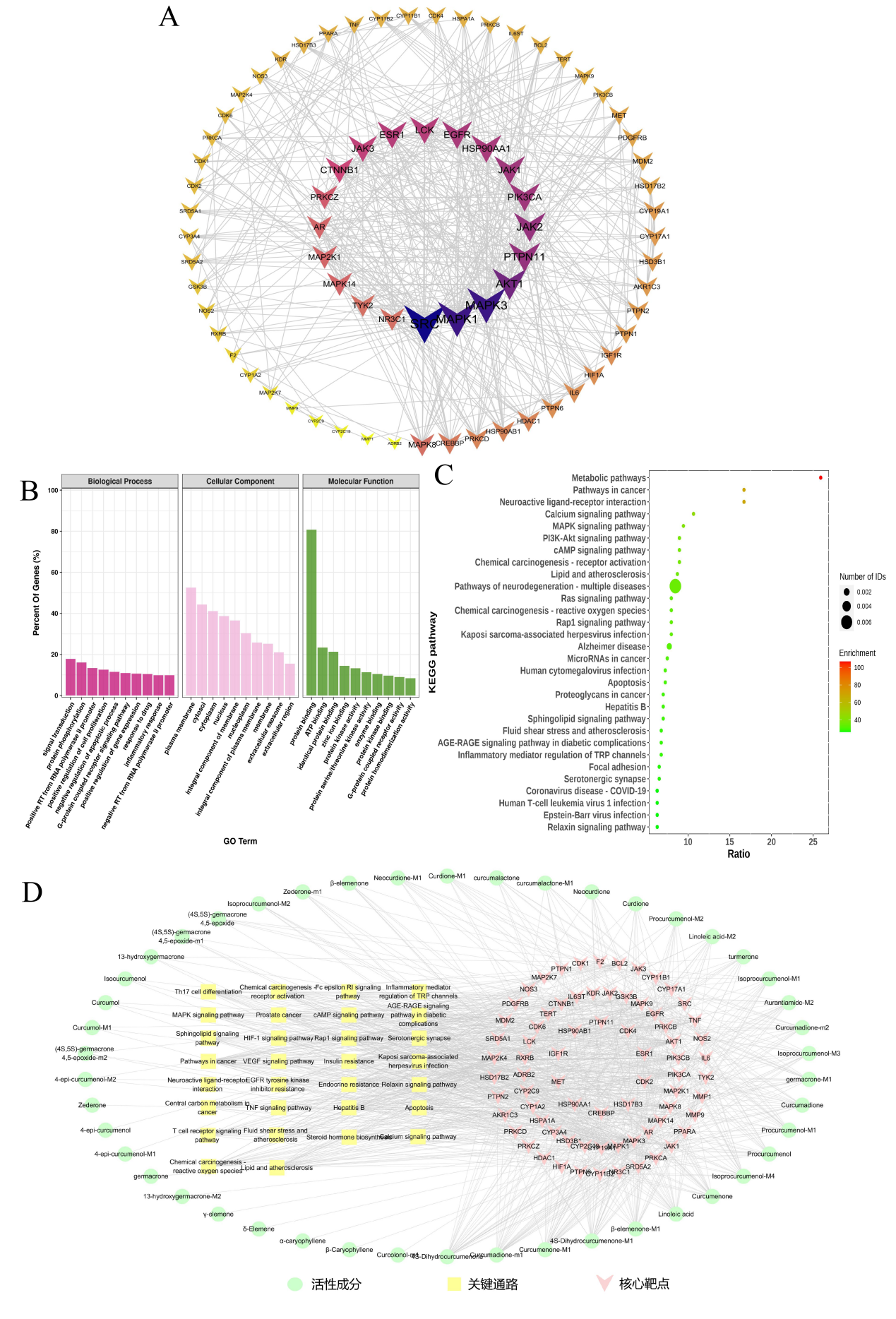


**Fig. S3** Network pharmacology results of CW in the treatment of PD. (A) PPI network of core targets; (B) GO enrichment analysis; (C) KEGG pathway analysis; (D) Network of *active ingredients - core targets - critical pathways*.


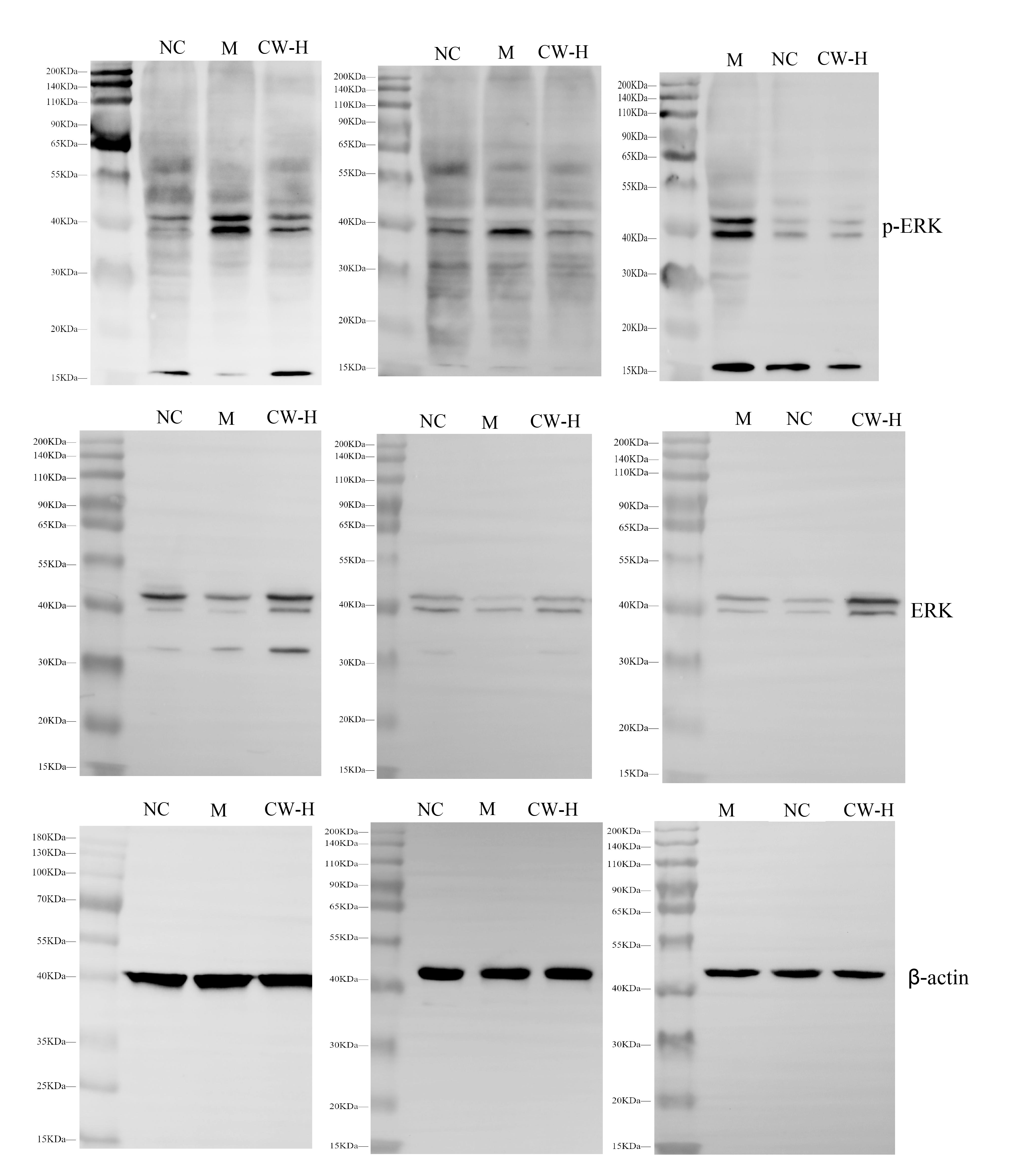


**Fig. S4** Raw data from the WB experiment


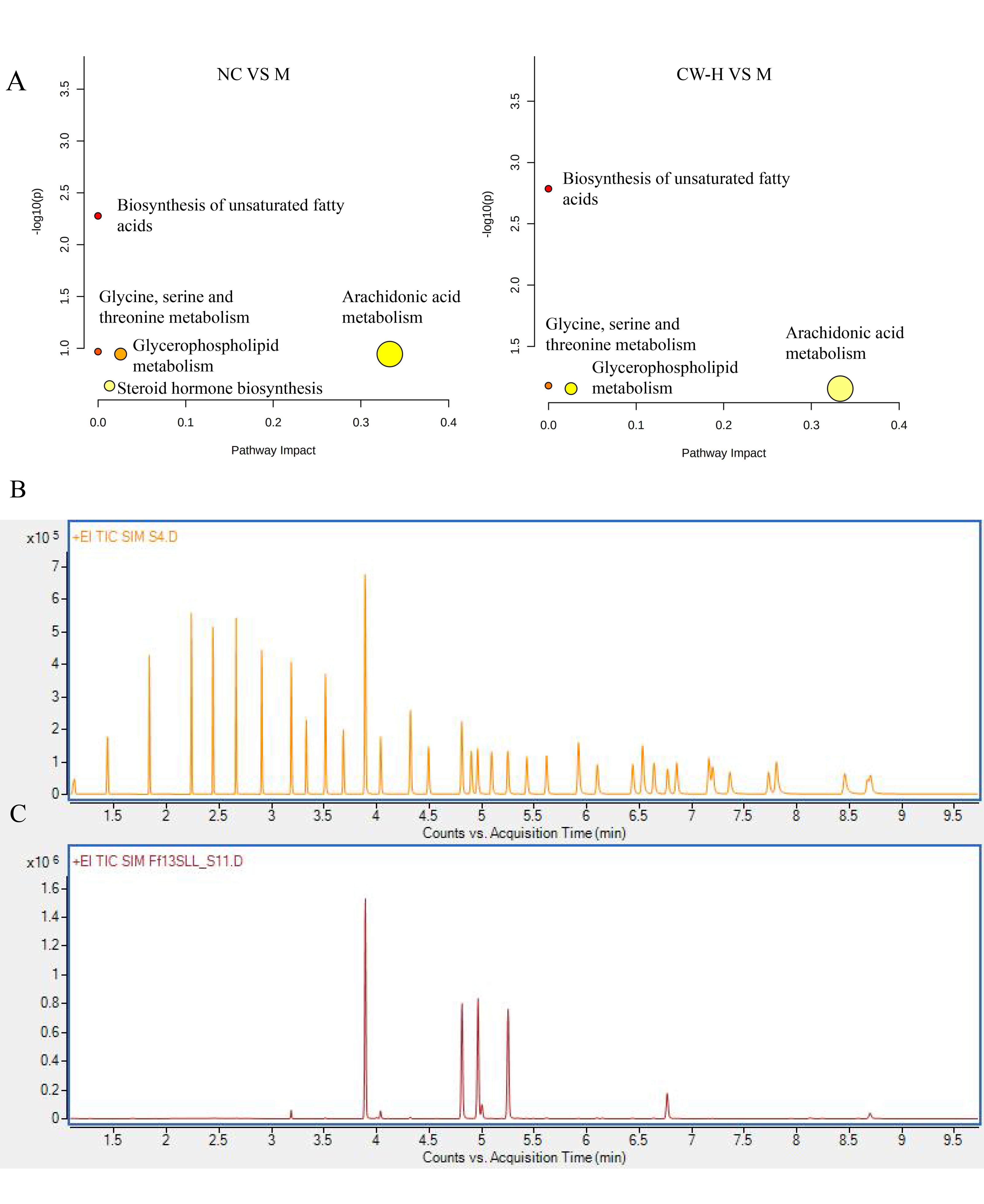


**Fig. S5** Study on serum non targeted metabonomics and fatty acid targeted metabonomics. (A) Analysis of differential metabolic pathways of serum samples from each group. NC-Normal control group, M-Model group, CW-H-CW high-dose group. (B) TIC plots of typical samples for fatty acid metabolism; (C) TIC plots of standards for fatty acid metabolism.
