# Supplementary material for: The therapeutic mechanism of Curcumae Radix against primary dysmenorrea based on 5-HTR/Ca2+/MAPK and fatty acids metabolomics
Source: Front Pharmacol. 2023 Mar 9;14:1087654. doi: 10.3389/fphar.2023.1087654 (PMC10034069; doi:10.3389/fphar.2023.1087654)
Supplement: Supplementary file 1 [file DataSheet1.zip › Supplemental materials/Supplemental materials-tableS1-S7.docx]

**Table S1** RT-qPCR primers for candidate genes

| Name | Genes | Primer name | Primer sequences (5’-3’) |
| --- | --- | --- | --- |
| β-actin | β-actin | β-actin-F | AGATGACCCAGATCATGTTTGAGA |
|  |  | β-actin-R | GCATGAGGGAGCGCGTAA |
| 5-hydroxytryptamine receptor 2A | 5-HTR2A | 5-HTR2A-F | TCCTTGTCATGCCTGTGTCC |
|  |  | 5-HTR2A-R | TGAATGGGGTTCTGGATGGC |
| Inositol 1,4,5-trisphosphate receptor | IP3R | IP3R-F | AGCTTGAACCAAGTCCACCC |
|  |  | IP3R-R | GTGGACCATTGCCAAAGCTG |
| Calmodulin | cALM | cALM-F | GCTACATCAGTGCGGCAGAA |
|  |  | cALM-R | TAGTTGACCTGTCCGTCTCC |
| Protein kinase C | PKC | PKC-F | TATGACCAAACACCCTGCCAA |
|  |  | PKC-R | CTCCTTTGCCGCACACTTTG |
| Extracellular signal-regulated kinase | ERK | ERK-F | GGTTGTTCCCAAACGCTGAC |
|  |  | ERK-R | ATACTGCTCCAGGTACGGGT |
| Note: *F* stood for forward primer and *R* stood for reverse primer. | | | |

**Table S2** Effect of extract of CW on contractile of the isolated uterus (‾x ± s, n ＝ 6)

| Evaluation indexs | Groups | Administration doses (g/L) | Blank curve | Model curve | After administration | Inhibition rate(%) |
| --- | --- | --- | --- | --- | --- | --- |
| Contractile tension (g) | B | 0.05 | 2.24 ± 0.93 | 6.06 ± 2.15^**^ | 2.48 ± 1.48^##^ | 59.08 |
|  | CW | 4.00 | 2.60 ± 0.88 | 7.16 ± 1.15^***^ | 6.91 ± 1.04 | 3.49 |
|  |  | 6.00 | 2.60 ± 0.88 | 7.16 ± 1.15^***^ | 6.50 ± 0.83 | 9.22 |
|  |  | 8.00 | 2.60 ± 0.88 | 7.16 ± 1.15^***^ | 5.66 ± 0.98^#^ | 20.95 |
| Contractile amplitude (g) | B | 0.05 | 8.48 ± 1.83 | 9.00 ± 1.05 | 4.18 ± 1.18^###^ | 53.56 |
|  | CW | 4.00 | 10.32 ± 1.28 | 13.18 ± 2.01^*^ | 11.54 ± 2.22 | 12.44 |
|  |  | 6.00 | 10.32 ± 1.28 | 13.18 ± 2.01^*^ | 9.10 ± 2.13^##^ | 30.96 |
|  |  | 8.00 | 10.32 ± 1.28 | 13.18 ± 2.01^*^ | 6.38 ± 1.31^###^ | 51.59 |
| Contractile frequency (Times/10 min) | B | 0.05 | 6.25 ± 2.09 | 6.42 ± 1.83 | 4.25 ± 1.33 | 33.80 |
|  | CW | 4.00 | 6.00 ± 1.26 | 6.58 ± 0.80 | 6.17 ± 0.82 | 6.23 |
|  |  | 6.00 | 6.00 ± 1.26 | 6.58 ± 0.80 | 5.50 ± 0.55 | 16.41 |
|  |  | 8.00 | 6.00 ± 1.26 | 6.58 ± 0.80 | 4.50 ± 0.77^##^ | 31.61 |
| Contractile activity (g×Times/10 min) | B | 0.05 | 52.94 ± 20.20 | 57.99 ± 21.18 | 17.38±8.98^###^ | 70.03 |
|  | CW | 4.00 | 62.01 ± 15.37 | 86.19 ± 12.87^**^ | 70.48±12.35 | 18.23 |
|  |  | 6.00 | 62.01 ± 15.37 | 86.19 ± 12.87^**^ | 50.18±13.54^###^ | 41.78 |
|  |  | 8.00 | 62.01 ± 15.37 | 86.19 ± 12.87^**^ | 28.48±6.07^###^ | 66.96 |
| Compared with blank curve, ^*^*p* < 0.05,^**^*p* < 0.01, ^***^*p* < 0.001; compared with model curve, ^#^*p* < 0.05, ^##^*p* < 0.01, ^###^*p* < 0.001.  CW, *Curcumae Radix* group; IBP, Ibuprofen positive control group. | | | | | | |

**Table S3** The information of serum components and metabolites of CW was characterized by UPLC-Q-TOF-MS/MS

| No. | Rt (min) | Assignedidentity | Metabolic pathways | Molecular formula | Molecular Mass (Da) | Adduct | Found at mass（Da) | PPM |
| --- | --- | --- | --- | --- | --- | --- | --- | --- |
| 1 | 5.91 | Zedoalactone A | parent | C_15_H_22_O_4_ | 266.1518 | [M+H]^+^ | 267.1592 | 0.50 |
| 2 | 5.91 | Zedoalactone C | parent | C_15_H_22_O_4_ | 266.1518 | [M+H]^+^ | 267.1592 | 0.50 |
| 3 | 7.06 | Curcolonol | parent | C_15_H_20_O_4_ | 264.1362 | [M+H]^+^ | 265.1436 | 0.80 |
| 4 | 6.53 | Curcolonol-M1 | Loss of O and O | C_15_H_20_O_2_ | 232.1500 | [M+H]^+^ | 233.1538 | 1.00 |
| 5 | 6.51 | Curcolonol-M2 | Loss of H-2O | C_15_H_22_O_3_ | 250.1600 | [M+H]^+^ | 251.1646 | 1.90 |
| 6 | 6.24 | Zederone | parent | C_15_H_18_O_3_ | 246.1256 | [M+H]^+^ | 247.1333 | 1.80 |
| 7 | 6.53 | Zederone-M1 | Loss of H-2O | C_15_H_20_O_2_ | 232.1500 | [M+H]^+^ | 233.1538 | 1.00 |
| 8 | 6.24 | curcolone | parent | C_15_H_18_O_3_ | 246.1256 | [M+H]^+^ | 247.1333 | 1.80 |
| 9 | 6.53 | curcolone-M1 | Loss of H-2O | C_15_H_20_O_2_ | 232.1500 | [M+H]^+^ | 233.1538 | 1.00 |
| 10 | 8.95 | γ-elemene | Ketone Formation | C_15_H_22_O | 218.1700 | [M+H]^+^ | 219.1746 | 1.30 |
| 11 | 8.95 | δ-Elemene | Ketone Formation | C_15_H_22_O | 218.1700 | [M+H]^+^ | 219.1746 | 1.30 |
| 12 | 8.95 | β-selinene | Ketone Formation | C_15_H_22_O | 218.1700 | [M+H]^+^ | 219.1746 | 1.30 |
| 13 | 8.95 | α-caryophyllene | Ketone Formation | C_15_H_22_O | 218.1700 | [M+H]^+^ | 219.1746 | 1.30 |
| 14 | 8.95 | β-Caryophyllene | Ketone Formation | C_15_H_22_O | 218.1700 | [M+H]^+^ | 219.1746 | 1.30 |
| 15 | 9.98 | Aurantiamide-M1 | Loss of O and C16H13NO2+Amine to Carboxylic Acid | C_10_H_12_O_2_ | 164.0800 | [M+H]^+^ | 165.091 | -0.20 |
| 16 | 7.14 | Aurantiamide-M2 | Loss of C9H10O+Oxidation | C_16_H_16_N_2_O_3_ | 284.1200 | [M+H]^+^ | 285.1233 | -2.20 |
| 17^※^ | 5.48 | Curcumenol | parent | C_15_H_22_O_2_ | 234.1620 | [M+H]^+^ | 235.1698 | 1.50 |
| 18 | 8.97 | Curcumenol-M1 | Loss of O | C_15_H_22_O | 218.1700 | [M+H]^+^ | 219.1743 | -0.30 |
| 19 | 8.72 | Procurcumenol | parent | C_15_H_22_O_2_ | 234.1620 | [M+H]^+^ | 235.17 | 3.10 |
| 20 | 9.91 | Procurcumenol-M1 | Hydrogenation | C_15_H_24_O_2_ | 236.1800 | [M+H]^+^ | 237.1851 | 0.80 |
| 21 | 8.97 | Procurcumenol-M2 | Loss of O | C_15_H_22_O | 218.1700 | [M+H]^+^ | 219.1743 | -0.30 |
| 22 | 5.46 | Curcumadione | parent | C_15_H_22_O_2_ | 234.1620 | [M+H]^+^ | 235.1696 | 1.40 |
| 23 | 9.87 | Curcumadione-M1 | Hydrogenation | C_15_H_24_O_2_ | 236.1600 | [M+H]^+^ | 237.1852 | 1.20 |
| 24 | 4.98 | Curcumadione-M2 | Desaturation | C_15_H_20_O_2_ | 232.1500 | [M+H]^+^ | 233.1538 | 0.70 |
| 25^※^ | 13.26 | Furanodiene | parent | C_15_H_20_O | 216.1514 | [M+H]^+^ | 217.1592 | 2.50 |
| 26 | 5.93 | Furanodiene-M1 | Loss of H-2O | C_15_H_22_ | 202.1700 | [M+H]^+^ | 203.1798 | 1.60 |
| 27 | 6.99 | Curcumenone | parent | C_15_H_22_O_2_ | 234.1620 | [M+H]^+^ | 235.1692 | -0.10 |
| 28 | 9.91 | Curcumenone-M1 | Hydrogenation | C_15_H_24_O_2_ | 236.1800 | [M+H]^+^ | 237.1851 | 0.80 |
| 29 | 8.72 | 13-hydroxygermacrone | parent | C_15_H_22_O_2_ | 234.1620 | [M+H]^+^ | 235.17 | 3.10 |
| 30 | 9.91 | 13-hydroxygermacrone-M1 | Hydrogenation | C_15_H_24_O_2_ | 236.1800 | [M+H]^+^ | 237.1851 | 0.80 |
| 31 | 8.97 | 13-hydroxygermacrone-M2 | Loss of O | C_15_H_22_O | 218.1700 | [M+H]^+^ | 219.1743 | -0.30 |
| 32 | 8.72 | (4S,5S)-germacrone 4,5-epoxide | parent | C_15_H_22_O_2_ | 234.1620 | [M+H]^+^ | 235.17 | 3.10 |
| 33 | 9.91 | (4S,5S)-germacrone 4,5-epoxide-M1 | Hydrogenation | C_15_H_24_O_2_ | 236.1800 | [M+H]^+^ | 237.1851 | 0.80 |
| 34 | 8.97 | (4S,5S)-germacrone 4,5-epoxide-M2 | Loss of O+Desaturation | C_15_H_22_O | 218.1700 | [M+H]^+^ | 219.1743 | -0.30 |
| 35 | 5.46 | 4-epi-curcumenol | parent | C_15_H_22_O_2_ | 234.1620 | [M+H]^+^ | 235.1698 | 1.40 |
| 36 | 4.98 | 4-epi-curcumenol-M1 | Desaturation | C_15_H_20_O_2_ | 232.1500 | [M+H]^+^ | 233.1538 | 0.70 |
| 37 | 5.5 | 4-epi-curcumenol-M2 | Desaturation to Carboxylic Aclid | C_15_H_20_O_4_ | 264.1400 | [M+H]^+^ | 265.1436 | 0.60 |
| 38 | 7.9 | 4-epi-curcumenol-M3 | Loss of O | C_15_H_22_O | 218.1700 | [M+H]^+^ | 219.1745 | 0.80 |
| 39 | 12.68 | Isoprocurcumenol | parent | C_15_H_22_O_2_ | 234.1620 | [M+H]^+^ | 235.1698 | 1.50 |
| 40 | 9.87 | Isoprocurcumenol-M1 | Hydrogenation | C_15_H_24_O_2_ | 236.1800 | [M+H]^+^ | 237.1852 | 1.20 |
| 41 | 7.9 | Isoprocurcumenol-M2 | Loss of O | C_15_H_22_O | 218.1700 | [M+H]^+^ | 219.1745 | 0.80 |
| 42 | 6.96 | Isoprocurcumenol-M3 | Internal Hydrolysis | C_15_H_24_O_3_ | 252.1700 | [M+H]^+^ | 253.1799 | 0.20 |
| 43 | 4.98 | Isoprocurcumenol-M4 | Desaturation | C_15_H_20_O_2_ | 232.1500 | [M+H]^+^ | 233.1538 | 0.70 |
| 44 | 12.53 | turmerone | parent | C_15_H_22_O | 218.1671 | [M+H]^+^ | 219.1744 | 0.20 |
| 45 | 11.62 | β-elemenone | parent | C_15_H_22_O | 218.1671 | [M+H]^+^ | 219.1743 | -0.10 |
| 46 | 9.18 | β-elemenone-M1 | Oxidation | C_15_H_22_O_2_ | 234.1600 | [M+H]^+^ | 235.1695 | 1.00 |
| 47 | 10.51 | Curcumol | parent | C_15_H_24_O_2_ | 236.1800 | [M+H]^+^ | 237.1858 | 3.70 |
| 48 | 5.48 | Curcumol-M1 | Desaturation | C_15_H_22_O_2_ | 234.1600 | [M+H]^+^ | 235.1698 | 1.50 |
| 49^※^ | 10.51 | Curdione | parent | C_15_H_24_O_2_ | 236.1776 | [M+H]^+^ | 237.1858 | 3.70 |
| 50 | 5.48 | Curdione-M1 | Desaturation | C_15_H_22_O_2_ | 234.1600 | [M+H]^+^ | 235.1696 | 1.50 |
| 51 | 10.51 | Neocurdione | parent | C_15_H_24_O_2_ | 236.1776 | [M+H]^+^ | 237.1858 | 3.70 |
| 52 | 5.48 | Neocurdione-M1 | Desaturation | C_15_H_22_O_2_ | 234.1600 | [M+H]^+^ | 235.1696 | 1.50 |
| 53 | 9.91 | 4S-Dihydrocurcumenone | parent | C_15_H_24_O_2_ | 236.1776 | [M+H]^+^ | 237.1851 | 0.80 |
| 54 | 5.48 | 4S-Dihydrocurcumenone-M1 | Desaturation | C_15_H_22_O_2_ | 234.1600 | [M+H]^+^ | 235.1696 | 1.50 |
| 55 | 9.91 | curcumalactone | parent | C_15_H_24_O_2_ | 236.1776 | [M+H]^+^ | 237.1851 | 0.80 |
| 56 | 5.48 | curcumalactone-M1 | Desaturation | C_15_H_22_O_2_ | 234.1600 | [M+H]^+^ | 235.1696 | 1.50 |
| 57^※^ | 21.97 | Isocurcumenol | parent | C_15_H_22_O_2_ | 234.1620 | [M+H]^+^ | 235.1699 | 2.90 |
| 58 | 8.97 | Isocurcumenol-M1 | Loss of O | C_15_H_22_O | 218.1700 | [M+H]^+^ | 219.1743 | -0.30 |
| 59^※^ | 12.53 | germacrone | parent | C_15_H_22_O | 218.1671 | [M+H]^+^ | 219.1744 | 0.20 |
| 60 | 9.18 | germacrone-M1 | Oxidation | C_15_H_22_O_2_ | 234.1600 | [M+H]^+^ | 235.1695 | 1.00 |
| 61 | 13.79 | Isobutyl phthalate | parent | C_16_H_22_O_4_ | 278.1518 | [M+H]^+^ | 279.1598 | 2.40 |
| 62^※^ | 8.95 | β-Elemene | Ketone Formation | C_15_H_22_O | 218.1700 | [M+H]^+^ | 219.1746 | 1.30 |
| 63 | 19.34 | Linoleic acid | parent | C_18_H_32_O_2_ | 280.2402 | [M+H]^+^ | 281.2482 | 0.90 |
| 64 | 21.1 | Linoleic acid-M1 | Loss of O+Hydrogenation | C_18_H_34_O | 266.2600 | [M+H]^+^ | 267.2667 | 1.90 |
| 65 | 17.89 | Linoleic acid-M2 | Loss of O | C_18_H_32_O | 264.2500 | [M+H]^+^ | 265.2528 | 0.70 |
| 66 | 5.93 | Linoleic acid-M3 | Demethylation to Carboxylic Aclid | C_18_H_30_O_4_ | 310.2100 | [M+H]^+^ | 311.2214 | -0.90 |

^※^Identification with reference standards.

**Table S4** Identification of 29 differential metabolites from serum samples

| No. | HMDB ID | Mass（Da) | Molecular Formula | Name | Adducts | T_a_ | T_b_ |
| --- | --- | --- | --- | --- | --- | --- | --- |
| 1 | HMDB0000097 | 104.10641 | C_5_H_13_NO | Choline | [M+H]^+^ | ↓ | ↓ |
| 2 | HMDB0062769 | 114.09114 | C_6_H_11_NO | Epsilon caprolactam | [M+H]^+^ | — | — |
| 3 | HMDB0003411 | 116.07024 | C_5_H_9_NO_2_ | D-Pyrrolidine-2-carboxylic acid | [M+H]^+^ | — | — |
| 4 | HMDB0003355 | 118.08583 | C_5_H_11_NO_2_ | 5-Aminovaleric acid | [M+H]^+^ | — | — |
| 5 | HMDB0001406 | 123.0548 | C_6_H_6_N_2_O | Nicotinamide | [M+H]^+^ | — | — |
| 6 | HMDB0000716 | 130.0854 | C_6_H_11_NO_2_ | L-Pipecolic acid | [M+H]^+^ | — | — |
| 7 | HMDB0013773 | 132.10196 | C_6_H_13_NO_2_ | D-Leucine | [M+H]^+^ | — | — |
| 8 |  | 132.07606 | C_4_H_9_N_3_O_2_ | 3-Guanidinopropionic acid | [M+H]^+^ | ↓ | — |
| 9 | HMDB0000696 | 150.05821 | C_5_H_11_NO_2_S | L-Methionine | [M+H]^+^ | — | — |
| 10 | HMDB0014389 | 154.05886 | C_7_H_7_NO_3_ | Mesalamine | [M+H]^+^ | — | — |
| 11 |  | 159.0922 | C_10_H_10_N_2_ | β-Nicotyrine | [M+H]^+^ | — | — |
| 12 | HMDB0000062 | 162.11217 | C_7_H_15_NO_3_ | L-Carnitine | [M+H]^+^ | — | — |
| 13 | HMDB0004350 | 163.11625 | C_10_H_14_N_2_ | (-)-Anabasine | [M+H]^+^ | — | — |
| 14 | HMDB0000517 | 175.11976 | C_6_H_14_N_4_O_2_ | L-Arginine | [M+H]^+^ | — | — |
| 15 |  | 182.08169 | C_9_H_11_NO_3_ | Dl-Tyrosine | [M+H]^+^ | — | — |
| 16 | HMDB0000821 | 194.08091 | C_10_H_11_NO_3_ | N-Phenylacetylglycine | [M+H]^+^ | — | — |
| 17 | HMDB0032055 | 198.08498 | C_8_H_11_N_3_O_3_ | N-Acetyl-L-histidine | [M+H]^+^ | — | — |
| 18 | HMDB0000101 | 252.10761 | C_10_H_13_N_5_O_3_ | Deoxyadenosine | [M+H]^+^ | — | — |
| 19 | HMDB0011171 | 261.14427 | C_11_H_20_N_2_O_5_ | γ-Glu-Leu | [M+H]^+^ | — | — |
| 20 | HMDB0000207 | 283.25486 | C_18_H_34_O_2_ | Oleic acid | [M+H]^+^ | — | — |
| 21 | HMDB0001043 | 305.24885 | C_20_H_32_O_2_ | Arachidonic acid | [M+H]^+^ | ↓ | ↓ |
| 22 | HMDB0002183 | 329.24867 | C_22_H_32_O_2_ | Docosahexaenoic acid | [M+H]^+^ | ↓ | ↓ |
| 23 |  | 331.26411 | C_22_H_34_O_2_ | All-cis-4,7,10,13,16-docosapentaenoic acid | [M+H]^+^ | ↓ | ↓ |
| 24 |  | 331.28472 | C_19_H_38_O_4_ | Glycerol 1-hexadecanoate | [M+H]^+^ | ↓ | ↓ |
| 25 | HMDB0000015 | 347.22227 | C_21_H_30_O_4_ | Cortexolone | [M+H]^+^ | ↓ | — |
| 26 | HMDB0003540 | 348.08014 | C_10_H_14_N_5_O_7_P | 3'-Aenylic acid | [M+H]^+^ | — | ↓ |
| 27 |  | 365.10508 | C_12_H_22_O_11_ | β-Gentiobiose | [M+Na]^+^ | — | ↓ |
| 28 |  | 455.18865 | C_9_H_13_N_3_O_4_ | 2'-Deoxycytidine | [2M+H]^+^ | — | — |
| 29 | HMDB0001413 | 489.10488 | C_14_H_26_N_4_O_11_P_2_ | Citicoline | [M+H]^+^ | — | — |
| Note: *T_a_* represented the normal control group compared with the model group; *T_b_* represented the CW high-dose group compared with the model group. | | | | | | | |

**Table S5** Results in WB experiments

| Integrated density val | | | | | | |
| --- | --- | --- | --- | --- | --- | --- |
| No. | (1)p-ERK | (2)p-ERK | (3)p-ERK | (1)ERK | (2)ERK | (3)ERK |
| NC | 113275 | 130770 | 74964 | 186801 | 89052 | 50037 |
| M | 268211 | 280848 | 256301 | 101105 | 57616 | 112494 |
| CW-H | 139606 | 109476 | 70264 | 187294 | 86913 | 228366 |
| Indicators integrated density val/Internal reference integrated density val（ratio） | | | | | | |
| NC | 0.26 | 0.32 | 0.22 | 0.43 | 0.22 | 0.15 |
| M | 0.61 | 0.68 | 0.77 | 0.23 | 0.14 | 0.34 |
| CW-H | 0.32 | 0.27 | 0.21 | 0.43 | 0.21 | 0.68 |

**Table S6** Standard curve of mixed standard solution

| NO . | Name | Range (μg/mL) | Curve equation | R^2^ | Quantitative limit |
| --- | --- | --- | --- | --- | --- |
| 1 | C6:0 | 0.99-131.5 | y = 1262.156385 * x +4488.272697 | 0.996 | 0.99 |
| 2 | C8:0 | 0.99-131.5 | y = 1785.867948 * x +3263.443031 | 0.989 | 0.99 |
| 3 | C10:0 | 0.99-131.5 | y = 1853.797105 * x +3114.312667 | 0.990 | 0.99 |
| 4 | C11:0 | 0.99-131.5 | y = 1657.217647 * x +3680.395412 | 0.998 | 0.99 |
| 5 | C12:0 | 0.99-131.5 | y = 1721.060823 * x +2711.184638 | 0.989 | 0.99 |
| 6 | C13:0 | 0.99-131.5 | y = 1641.234681 * x +2419.467517 | 0.989 | 0.99 |
| 7 | C14:0 | 0.99-131.5 | y = 1558.267988 * x  +2062.441461 | 0.994 | 0.99 |
| 8 | C14:1 | 0.99-131.5 | y = 564.463448 * x  +668.967520 | 0.988 | 0.99 |
| 9 | C15:0 | 0.99-131.5 | y = 1565.948159 * x  +1717.848679 | 0.991 | 0.99 |
| 10 | C15:1 | 0.99-131.5 | y = 584.079822 * x  +638.764176 | 0.996 | 0.99 |
| 11 | C16:0 | 0.99-131.5 | y = 177.461964 * x  +409.226853 | 0.988 | 0.99 |
| 12 | C16:1 | 0.99-131.5 | y = 458.200131 * x  +469.806705 | 0.996 | 0.99 |
| 13 | C17:0 | 0.99-131.5 | y = 1391.236503 * x  +1143.981074 | 0.991 | 0.99 |
| 14 | C17:1 | 0.99-131.5 | y = 479.049546 * x  +338.899509 | 0.995 | 0.99 |
| 15 | C18:0 | 0.99-131.5 | y = 1318.406845 * x  +845.159956 | 0.992 | 0.99 |
| 16 | C18:1n9t | 0.99-131.5 | y = 435.333564 * x  +275.900536 | 0.996 | 0.99 |
| 17 | C18:1n9c | 0.99-131.5 | y = 472.490974 * x  +440.049415 | 0.996 | 0.99 |
| 18 | C18:2n6t | 0.99-131.5 | y = 485.736415 * x  +225.258336 | 0.992 | 0.99 |
| 19 | C18:2n6c | 0.99-131.5 | y = 504.359409 * x  +270.497381 | 0.993 | 0.99 |
| 20 | C20:0 | 0.99-131.5 | y = 1161.651467 * x  +551.381608 | 0.992 | 0.99 |
| 21 | C18:3n6 | 0.99-131.5 | y = 450.218254 * x  +249.558425 | 0.993 | 0.99 |
| 22 | C20:1 | 0.99-131.5 | y = 423.690292 * x  +183.708577 | 0.992 | 0.99 |
| 23 | C18:3n3 | 0.99-131.5 | y = 527.336675 * x  +267.753443 | 0.991 | 0.99 |
| 24 | C21:0 | 0.99-131.5 | y = 1141.836017 * x  +413.728367 | 0.992 | 0.99 |
| 25 | C20:2 | 0.99-131.5 | y = 387.426775 * x  +180.475162 | 0.985 | 0.99 |
| 26 | C22:0 | 0.99-131.5 | y = 1075.772304 * x  +332.723914 | 0.989 | 0.99 |
| 27 | C20:3n6 | 0.99-131.5 | y = 377.115856 * x  +113.356625 | 0.991 | 0.99 |
| 28 | C22:1n9 | 0.99-131.5 | y = 399.046674 * x  +117.836536 | 0.989 | 0.99 |
| 29 | C20:3n3 | 0.99-131.5 | y = 491.946498 * x  +175.959528 | 0.989 | 0.99 |
| 30 | C23:0 | 0.99-131.5 | y = 86.644300 * x  -42.788503 | 0.991 | 0.99 |
| 31 | C20:4n6 | 0.99-131.5 | y = 422.642725 * x  +178.101399 | 0.989 | 0.99 |
| 32 | C22:2 | 0.99-131.5 | y = 338.549522 * x  -325.972017 | 0.984 | 0.99 |
| 33 | C24:0 | 0.99-131.5 | y = 713.662124 * x +235.262852 | 0.980 | 0.99 |
| 34 | C20:5n3 | 0.99-131.5 | y = 486.714962 * x +177.381972 | 0.991 | 0.99 |
| 35 | C24:1 | 0.99-131.5 | y = 422.596645 * x +153.759990 | 0.990 | 0.99 |
| 36 | C22:6n3 | 0.99-131.5 | y = 408.733008 * x +143.963966 | 0.984 | 0.99 |

Table S7 Stability results of 36 medium and long chain FA in QC samples

| No. | Category 1 | Category 2 | RT (min) | Name | RSD (%) |
| --- | --- | --- | --- | --- | --- |
| 1 | Medium chain FA | SFA | 1.437 | C6:0 | 3.79 |
| 2 |  | SFA | 1.84 | C8:0 | 3.48 |
| 3 |  | SFA | 2.24 | C10:0 | 13.55 |
| 4 |  | SFA | 2.445 | C11:0 | 2.40 |
| 5 |  | SFA | 2.666 | C12:0 | 2.88 |
| 6 | Long chain FA | SFA | 2.913 | C13:0 | 6.65 |
| 7 |  | SFA | 3.19 | C14:0 | 2.85 |
| 8 |  | MUFA | 3.335 | C14:1 | 1.32 |
| 9 |  | SFA | 3.517 | C15:0 | 5.19 |
| 10 |  | MUFA | 3.688 | C15:1 | 0.50 |
| 11 |  | SFA | 3.335 | C16:0 | 2.33 |
| 12 |  | MUFA | 4.042 | C16:1 | 4.92 |
| 13 |  | SFA | 4.327 | C17:0 | 5.25 |
| 14 |  | MUFA | 4.498 | C17:1 | 4.26 |
| 15 |  | SFA | 4.814 | C18:0 | 3.87 |
| 16 |  | MUFA | 4.901 | C18:1n9t | 3.09 |
| 17 |  | MUFA | 4.962 | C18:1n9c | 3.87 |
| 18 |  | PUFA | 5.095 | C18:2n6t | 2.51 |
| 19 |  | PUFA | 5.251 | C18:2n6c | 2.40 |
| 20 |  | SFA | 5.924 | C20:0 | 1.55 |
| 21 |  | PUFA | 5.434 | C18:3n6 | 5.23 |
| 22 |  | MUFA | 6.103 | C20:1 | 1.52 |
| 23 |  | PUFA | 5.62 | C18:3n3 | 4.94 |
| 24 |  | SFA | 6.529 | C21:0 | 1.90 |
| 25 |  | PUFA | 6.437 | C20:2 | 5.04 |
| 26 |  | SFA | 7.16 | C22:0 | 2.69 |
| 27 |  | PUFA | 6.639 | C20:3n6 | 3.36 |
| 28 |  | MUFA | 7.361 | C22:1n9 | 1.02 |
| 29 |  | PUFA | 6.856 | C20:3n3 | 6.62 |
| 30 |  | SFA | 7.73 | C23:0 | 11.76 |
| 31 |  | PUFA | 6.772 | C20:4n6 | 5.18 |
| 32 |  | PUFA | 7.704 | C22:2 | 5.96 |
| 33 |  | SFA | 8.449 | C24:0 | 4.62 |
| 34 |  | PUFA | 7.202 | C20:5n3 | 5.54 |
| 35 |  | MUFA | 8.666 | C24:1 | 4.96 |
| 36 |  | PUFA | 8.704 | C22:6n3 | 6.02 |
